# CD8 T Cell Memory to a Viral Pathogen Requires Trans Cosignaling between HVEM and BTLA

**DOI:** 10.1371/journal.pone.0077991

**Published:** 2013-10-29

**Authors:** Rachel Flynn, Tarun Hutchinson, Kenneth M. Murphy, Carl F. Ware, Michael Croft, Shahram Salek-Ardakani

**Affiliations:** 1 Department of Pathology, Immunology & Laboratory Medicine, University of Florida, Gainesville, Florida, United States of America; 2 Howard Hughes Medical Institute, Washington University School of Medicine, St. Louis, Missouri, United States of America; 3 La Jolla Institute for Allergy and Immunology, Divisions of Immune Regulation, San Diego, California, United States of America; 4 Laboratory of Molecular Immunology, Infectious and Inflammatory Diseases Center, Sanford Burnham Medical Research Institute, La Jolla, California, United States of America; MRC National Institute for Medical Research, United Kingdom

## Abstract

Defining the molecular interactions required to program activated CD8 T cells to survive and become memory cells may allow us to understand how to augment anti-viral immunity. HVEM (herpes virus entry mediator) is a member of the tumor necrosis factor receptor (TNFR) family that interacts with ligands in the TNF family, LIGHT and Lymphotoxin-α, and in the Ig family, B and T lymphocyte attenuator (BTLA) and CD160. The Ig family members initiate inhibitory signaling when engaged with HVEM, but may also activate survival gene expression. Using a model of vaccinia virus infection, we made the unexpected finding that deficiency in HVEM or BTLA profoundly impaired effector CD8 T cell survival and development of protective immune memory. Mixed adoptive transfer experiments indicated that BTLA expressed in CD8α+ dendritic cells functions as a trans-activating ligand that delivers positive co-signals through HVEM expressed in T cells. Our data demonstrate a critical role of HVEM-BTLA bidirectional cosignaling system in antiviral defenses by driving the differentiation of memory CD8 T cells.

## Introduction

An effective immune response to acute virus infections relies on the ability of a CD8+ T cells to quickly generate an expanded population of effector or cytotoxic T lymphocytes [1], [2], [3]. For long-term protection, part of the antigen-specific effector T cell pool must be retained as memory cells [4], [5], [6]. Defining the signals that control effective memory responses has broad implications for vaccine design and in the management of adverse immune reactions.

The fate of T cells after TCR engagement is influenced by both positive (costimulatory) and negative (coinhibitory) signals that can either amplify or limit T cell function. This regulation is provided through multiple spatially and temporally regulated interactions between receptors on T cells and their soluble or membrane-bound ligands expressed on antigen-presenting cells (APC) such as dendritic cells (DC cells) or B cells. Members of the tumor-necrosis-factor receptor (TNFR)/TNF superfamily have become recognized for their ability to stimulate T cells and provide co-signals that promote T cell clonal expansion and long-term survival. This includes the interactions of OX40 with OX40L, CD27 with CD70, TNFR with TNF, GITR with GITRL, CD30 with CD30L, and 4-1BB with 4-1BBL [7], [8], [9], [10], [11]. In addition to molecules in the TNFR/TNF superfamily, there are also other receptor-ligand pairs in divergent families that are further key positive regulators of T cells, including some of the Ig/CD28 superfamily, such as interactions of CD28 with B7.1 and B7.2 and ICOS with ICOSL [12]. These molecules have been proposed to either act together, or to act at different times in a temporal sequence, to sustain long-term protective immune responses. We also know that there are several suppressive or coinhibitory receptor-ligands pairs that directly oppose the costimulatory interactions described above, including molecules in the TNFR/TNF superfamily such as the death receptors Fas and TRAILR, and others in the Ig superfamily such as CTLA4, PD-1, B and T lymphocyte attenuator (BTLA), and CD160 [12],[13],[14],[15],[16],[17].

An important crosstalk between these co-signaling superfamilies occurs in the engagement of the herpesvirus entry mediator (HVEM, TNFRSF14) with the B and T lymphocyte attenuator (BTLA) [15], [17], [18], [19]. As the name suggests, HVEM was originally discovered because of its ability to bind to HSV viral glycoprotein D (gD) [20], which facilitates virus entry into host cells [21]. Subsequently, the TNF family ligand LIGHT (Lymphotoxins, Inducible, competes with HSV Glycoprotein D for HVEM, expressed by T cells) was identified as a binding partner for HVEM [22], [23], [24]. Ligation of HVEM on T cells by membrane-bound LIGHT delivers positive co-signals through HVEM that promote T cell survival, in part, by initiating activation of pro-survival transcription factors NF-kB and AP-1 [24]. By contrast, HVEM engagement of BTLA activates inhibitory signaling in T cells through recruitment of SHP-1 and SHP-2 phosphatases, which attenuate tyrosine kinases activated by TCR antigen recognition [25], [26]. Consistent with a proposed role as an inhibitory co-signaling receptor, BTLA-deficient T cells show increased proliferation [25], [27], [28], and BTLA-knockout mice have enhanced susceptibility to autoimmune disease and increased inflammatory responses [15], [25], [27], [29], [30], [31], [32], [33], [34]. Evidence gathered in simple mouse systems that involve priming with non-replicating antigen in an artificial inflammatory environment indicated that inhibitory signaling initiated through the HVEM-BTLA pathway predominantly proceeds in a unidirectional fashion, with HVEM activating inhibitory trans-signaling in adjacent BTLA expressing T cells [29], [35]. To add further to the complexity of this co-signaling system, recent studies have suggested that BTLA may also, under certain conditions, transmit positive co-signals into effector T cells that promote their survival [29], [34], [36]. This is consistent with the fact that in addition to the ITIM motifs, the BTLA intracellular domain has a conserved membrane-proximal tyrosine containing YDND motif, which binds to growth receptor bound 2 (Grb-2) and interacts with the p85 subunit of phosphatidylinositol 3-kinese (PI3K) [37]. Since BTLA binds exclusively to HVEM, this further suggests that HVEM can act as both an inhibitory and stimulatory ligand for BTLA. However, it is not clear how the specific physiological context of ligand-receptor engagement alters signaling activity of HVEM, either as a ligand or as a receptor.

Using an experimental vaccinia virus (VACV) infection in mice, we could specifically distinguish between HVEM and BTLA interactions that positively or negatively regulate anti-viral CD8 T cell responses. Unexpectedly, we observed that HVEM- and BTLA-deficient mice have a severe defect in mounting a protective CD8 T cell response against VACV. Mixed adoptive transfer experiments indicated that T cell-autonomous expression of HVEM but not BTLA is necessary for continued survival of virus-specific effector CD8 T cells and optimal generation of memory. Similar to CD8 T cells lacking HVEM, WT CD8 T cells failed to accumulate effectively in a BTLA-deficient environment upon viral challenge. Likewise, WT CD8 T cells co-cultured with VACV-infected BTLA^−/−^ CD8α+ DCs failed to survive and accumulate over time. These observations illustrate that HVEM and BTLA mediate cosignaling in *trans* between DC and CD8 cells required for immune memory.

## Results

### HVEM Expression After Vaccinia Virus Infection

**Figure 1:**
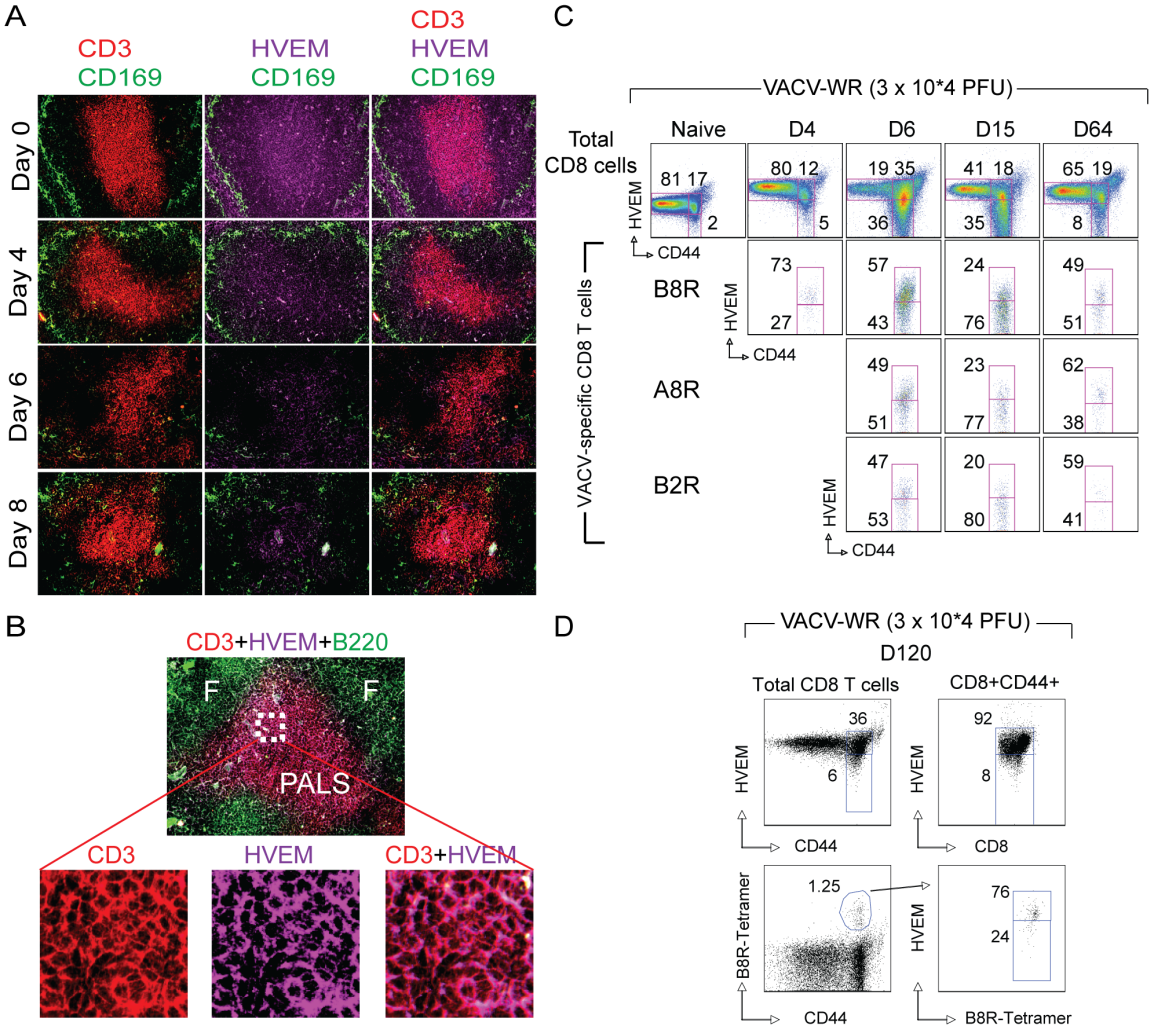
Localization and kinetics of HVEM expressing cells in the spleen of VACV infected mice. (A–D) Groups of C57BL/6 wild-type (WT) mice were infected i.p. with VACV-WR (3×10^4^ PFU/mouse). Uninfected (naïve) mice were used as controls. (A) Frozen sections of naïve (day 0), day 4, day 6 and day 8 VACV infected mice were stained with rat anti-mouse CD3-PE, HVEM-APC and CD169-FITC antibodies. The images were captured by 20× objective using EVOS *fl* inverted microscope. The micrographs arranged vertically in 1^st^, 2^nd^, and 3^rd^ column (left to right), showing localization of CD3 (red), HVEM (purple) and CD3+HVEM expression in the splenic white pulp respectively. CD169 (green) was used to identify splenic marginal zones. (B) B cell follicles (f) identified by B220 (green channel), perilymphatic sheath (PALS) identified by CD3 (red channel) and co-localization of HVEM and CD3 antibodies in PALS region of white pulp. On days 4, 6, 15, 64 (C), and day 120 (D) postinfection splenocytes were harvested and stained for CD8, CD44, VACV-specific tetramers (B8R, A8R, or B2R), and HVEM. Representative plots of HVEM staining on total CD8 T cells and tetramer (B8R, A8R, and B2R) positive cells after gating on CD8 cells. The numbers in each plot indicate the percentage of total CD8 T cells (CD44_low_ and CD44_high_) or tetramer-positive cells that stained for HVEM.

Initially we examined the localization of HVEM+ cells in the spleen of wild-type (WT) B6 mice infected with the mouse adapted vaccinia virus Western Reserve (VACV-WR) strain. Frozen spleen sections were stained with anti-CD3, anti-B220, anti-CD169 (MOMA), and anti-HVEM on days 0 (naïve), 4, 6, and 8 postinfection (PI) with VACV-WR and examined by microscopy (**Figure 1**). Splenic architecture is organized into distinct compartments (**Figure S1**). The white pulp (WP) includes the B cell follicles and a T cell area, the periarteriolar lymphoid sheath (PALS). The red pulp (RP) is a blood-filled space between each WP lymphoid follicle and the next. The marginal zone (MZ) separates the WP from the RP. In uninfected mice, HVEM+ cells were readily detectable in all areas of the WP and the RP (**Figure 1A**). Large numbers of HVEM+ T cells could be detected as clusters, primarily in the PALS of the splenic WP (**Figure 1B**). Starting at 4 days PI, a substantial decrease in HVEM+ cells was noted, with the majority of the positive cells now located in the PALS. However, by day 8 there was also a marked decrease in the proportion of HVEM positive lymphocytes in the PALS (**Figure 1A**).

Next, the surface expression of HVEM was monitored on total CD8 and virus-specific CD8 T cells by multi-parameter flow cytometry. We made use of H-2K^b^-tetramers containing the immunodominant B8R (20–27; TSYKFESV) and two subdominant A8R (189–196; ITYRFYLI) and B2R (54–62; YSQVNKRYI; H-2D^b^) VACV peptide epitopes to identify virus-specific CD8 T cells [38], [39]. In uninfected mice, HVEM was constitutively expressed at high levels on naïve (CD44^low^) CD8 T cells whereas primed (CD44^high^) T cells had slightly lower expression (**Figure 1C**). At the acute stage of the infection (between days 4 and 6), approximately 30–50% of all tetramer^+^ CD8 T cells in the spleen had down-regulated HVEM (**Figure 1C**). CD8^+^HVEM^–^ cells exclusively resided in the CD44^high^ T cell subset in the spleen. At day 15 after primary VACV-WR infection a significant number of tetramer^+^CD8^+^ T cells in the spleen remained HVEM^low^ and this still held true through the memory stage (Day 64). During the ‘late’ memory stage (day 120) the majority of tetramer^+^ CD8 T cells were HVEM positive and they had equivalent levels of HVEM compared to naïve (CD44^low^) cells (**Figure 1D**).

**Figure 2:**
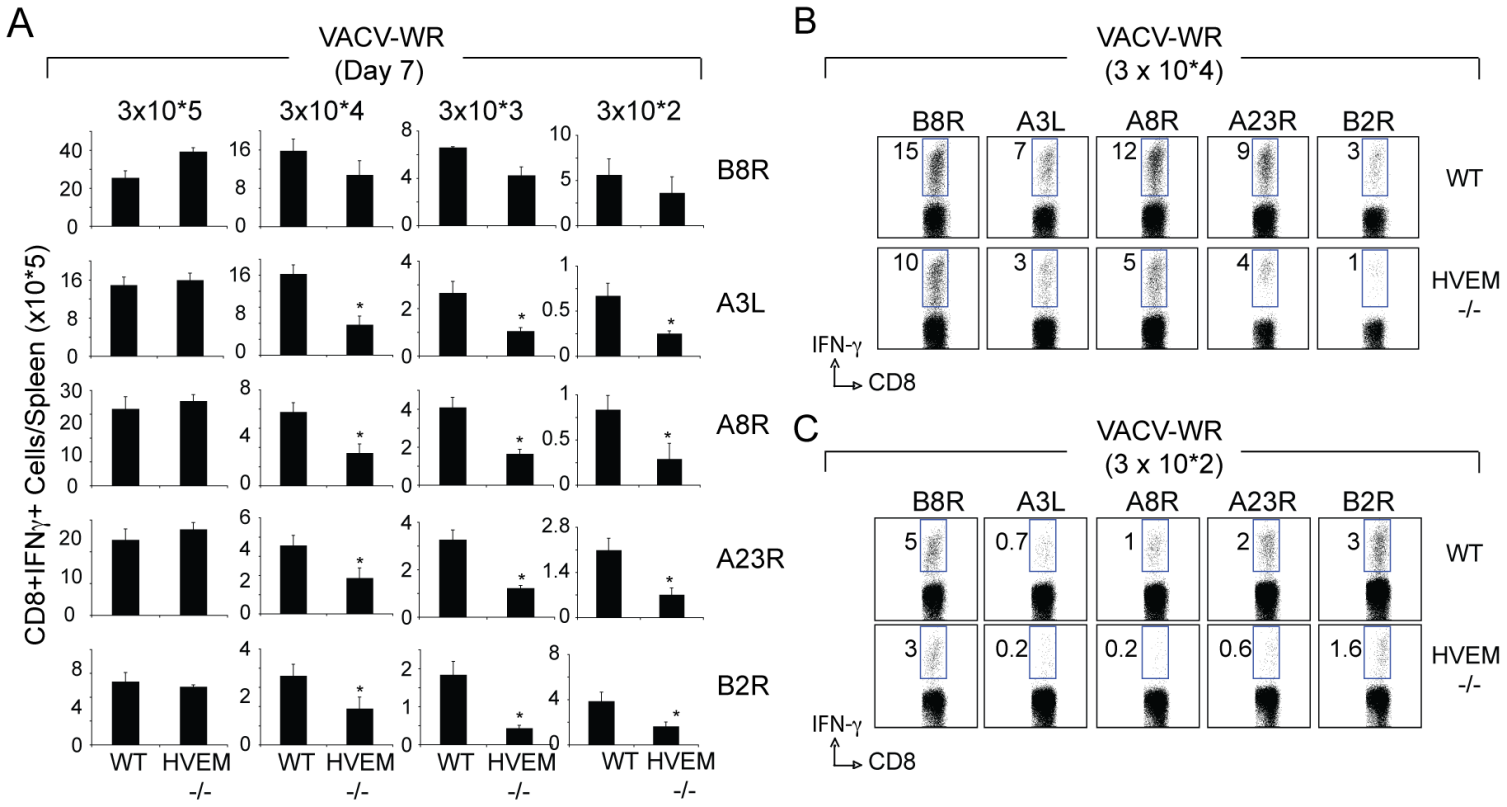
HVEM is required for optimal accumulation of virus-specific effector CD8 T cells directed against subdominant but not dominant VACV epitopes. Groups of C57BL/6 WT or HVEM-deficient (HVEM^−/−^) mice were infected i.p. with the indicated inoculums of VACV-WR. (A) On day 7-post infection, IFN-γ-secreting CD8 cells were assessed by intracellular cytokine staining after stimulation with the indicated VACV peptides (B8R, A3L, A8R, A23R, or B2R). Total numbers ± SEM of CD8^+^CD62L_low_IFN-γ^+^ T cells per spleen from four individual mice. *p<0.05 (WT vs HVEM^−/−^). Similar results were obtained in three separate experiments. Representative plots of IFN-γ staining in gated CD8 T cells after infection with 3×10^4^ PFU VACV-WR (B) or 3×10^2^ PFU VACV-WR (C). Numbers indicate the percentage of CD8^+^IFN-γ- positive cells. Similar results were obtained in three separate experiments.

To extend these observations, naïve (CD44^low^) OT-I CD8 T cells expressing a transgenic TCR (Vα_2_Vβ_5_) specific for H-2K^b^/OVA_257–264_ were transferred into WT mice, and HVEM expression was monitored after infection with recombinant VACV-WR expressing the full length OVA protein (rVACV-WR-OVA) (**Figure 2**). The transferred T cells were followed by the expression of the CD45.2 marker following transfer into CD45.1 congenic recipients. The kinetics of HVEM down regulation in OT-I cells was considerably faster than that on endogenous cells (CD45.2-negative). However, by day 54 both populations had fully re-expressed cell surface HVEM expression.

### HVEM Controls the Magnitude of Expansion of Effector CD8 T cells in Response to VACV Infection

The localization and kinetics of HVEM expression on CD8 T cells suggested that HVEM might play a role in primary expansion and effector function of VACV-specific CD8 T cells. To examine this, we infected WT and HVEM−/− mice with VACV-WR and monitored the generation of CD8 T cells specific for the dominant, B8R, and four subdominant (A3L, A8R, A23R, and B2R) MHC Class I-restricted VACV epitopes. Effector T cells were assessed by intracellular IFN-γ staining. These T cells account for up to 70% of the total VACV CD8 response [38], [39]. A high inoculum of VACV (3×10^5^ PFU; **Figure 2A**) in HVEM−/− mice induced primary CD8 T cell responses comparable to wild type, not only against B8R, but also against all subdominant epitopes examined. However, a strong role for HVEM was observed when the viral inoculum was lowered. The response of CD8 T cells to the subdominant VACV epitopes were reduced by as much as 75% in both the percentage and absolute numbers of T cells in HVEM−/− mice (**Figure 2A–C**). These results reveal that during the acute phase of infection HVEM plays an important role in generating large pools of VACV-specific effector CD8 T cells directed against subdominant but not the dominant epitope. Notably, the use of HVEM to drive enhanced CD8 T cell priming was closely related to the extent of exposure to viral inoculum.

**Figure 3:**
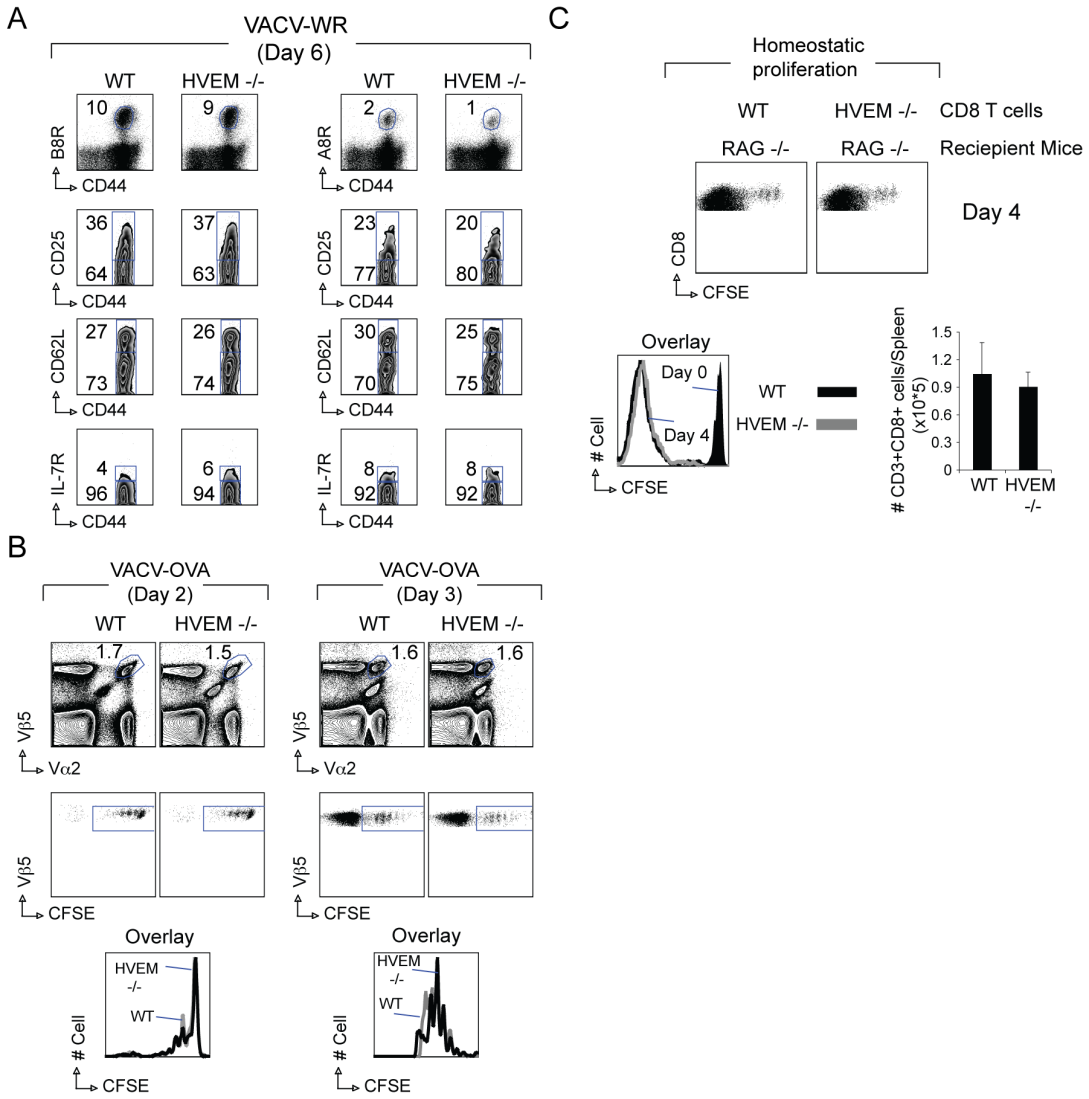
HVEM-deficient CD8 T cells have intact activation and early division in response to VACV infection or lymphopenic environment. (A) WT or HVEM^−/−^ mice were infected i.p. with VACV-WR (3×10^4^ PFU/mouse). On day 6-post infection, splenocytes were harvested and stained for CD8, CD44, and B8R or A8R tetramer. *Top panel*, Representative plots of tetramer staining, gating on CD8 cells. Percentages of CD44^high^ expressing B8R-tetramer (*left panels*) or A8R-tetramer (*right panels*) positive CD8 T cells are indicated. CD8 T cell activation was assessed by up-regulation of CD25, and down-regulation of CD62L and IL-7R (CD127) on tetramer-positive CD44^high^ cells. Naive (CD44^low^ tetramer-negative) CD8 T cells were used as controls. Percentages that stained positive for each marker are indicated. (B) One×10^5^ WT or HVEM −/− CFSE-labeled OT-I CD8 T cells were adoptively transferred into naïve WT mice. One day later, mice were infected i.p. with VACV-WR-OVA. After 2 or 3 days, OT-I CD8 T cells were analyzed. *Top*, Representative contour plots of co-staining for Vα2 and Vβ5 after gating on CD8^+^ T cells. Numbers indicate the percentage of CD8^+^Vα2^+^Vβ5^+^-positive cells. *Middle*, Representative dot plots of CFSE dilution after gating on CD8^+^Vα2^+^Vβ5^+^ cells. *Bottom*, Representative histogram of CFSE dilution gating on CD8^+^Vα2^+^Vβ5^+^ cells. (C) One×10^5^ highly purified CFSE-labeled polyclonal WT or HVEM^−/−^ CD8 T cells were adoptively transferred into RAG^−/−^ mice. After 4 days, splenocytes were harvested and stained with anti-CD3, anti-CD8, and anti-CD44. *Top*, Representative dot plots of CFSE dilution after gating on CD3^+^CD8^+^ cells. *Bottom*, Representative histogram of CFSE dilution gating on CD3^+^CD8^+^ cells. Results are the mean number ± SEM (*n* = 4 mice/group) from one experiment. Similar results were obtained in two separate experiments.

The initial activation state of VACV-specific CD8 T cells was largely unaffected in HVEM−/− mice as measured by expression of CD44 and CD25 (**Figure 3A**). Moreover, the down regulation of CD62L and IL7Rα in either B8R or A8R specific T cells was equivalent indicating HVEM is not essential for the initial activation of CD8 T cells.

To seek direct evidence on whether HVEM was required for the first rounds of cell division we employed two separate strategies. In our first approach, naïve WT or HVEM−/− OT-I CD8 T cells were labeled with carboxyfluorescein diacetate-succinimidyl ester (CFSE) and transferred into naive WT B6 mice (**Figure 3B**). One day later all recipient mice were infected with rVACV-WR-OVA as control. Challenge with PBS did not result in division or expansion of either WT or HVEM−/− OT-I cells (not shown). After infection with rVACV-WR-OVA recovered HVEM−/− OT-I cells displayed the same division profile as their WT counterparts (**Figure 3B**), and the same number of cells accumulated in the spleen at 48 and 72 hr postinfection (not shown).

In our second approach we assessed the requirement for intrinsic HVEM signals in the acute homeostasis-driven proliferation of naive polyclonal CD8 T cells in lymphopenic hosts (**Figure 3C**). Small numbers of purified CFSE-labeled CD8 T cells from WT and HVEM−/− mice were transferred into non-irradiated Rag −/− hosts and their capacity to divide and accumulate in the spleen were determined over a four day period. By day one after transfer, the extent of proliferation as measured by loss of CFSE signal was similar for both WT and HVEM−/− CD8 T cells transferred into Rag −/− hosts (not shown). By day four after transfer, all donor WT CD8 T cells divided, and many underwent more than eight rounds of division (**Figure 3C**). Again HVEM−/− CD8 T cells mounted a normal response and similar numbers were recovered from the spleen as compared with WT cells (**Figure 3C**). Thus, direct HVEM signaling in CD8 T cells is not essential for induction of T cell division and accumulation early in response to either VACV infection or homeostatic-driven proliferation.

### Impaired Generation of Memory CD8 T cells in the Absence of HVEM

**Figure 4:**
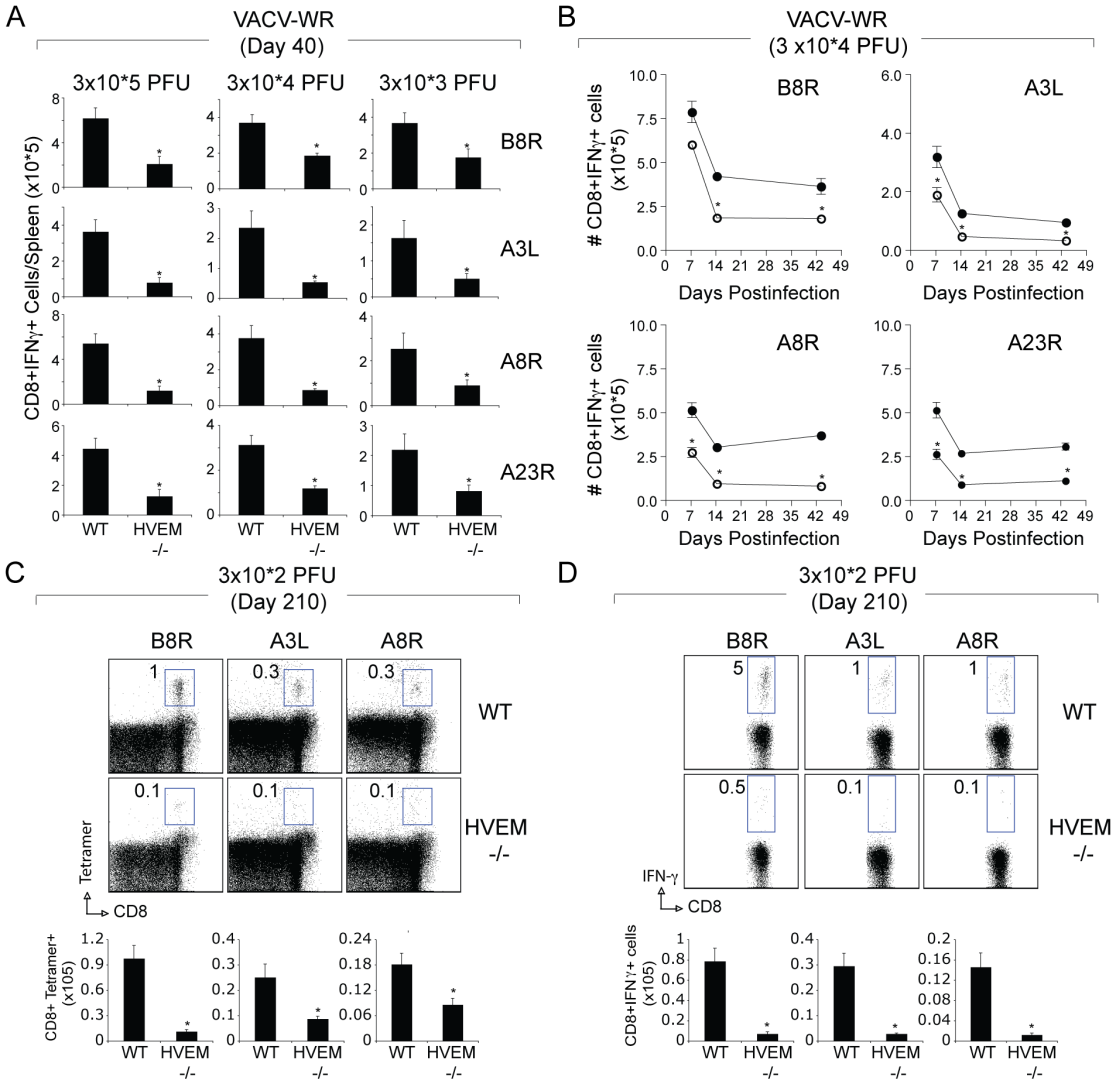
Impaired generation of CD8 memory cells to both dominant and subdominant VACV epitopes in HVEM-deficient mice. Groups of C57BL/6 WT or HVEM-deficient (HVEM^−/−^) mice were infected i.p. with the indicated inoculums of VACV-WR. At day 40 (A, B) day 7 (B), day 14 (B), or day 210 (C and D), splenocytes were harvested and stimulated with VACV peptides as indicated and CD8 T cell number and functionality was assessed by tetramer and intracellular IFN-γ staining. (A and B) Total numbers of CD8^+^IFN-γ^+^cells per spleen. Results are mean number ± SEM (*n* = 4 mice/group) from one experiment. (C) Representative plots of B8R, A3L, and A8R tetramer staining, gating on CD8 cells, are shown. Percentages of activated tetramer^+^ CD8 T cells (CD8^+^CD44^+^B8R^+^) are indicated. (D) Representative plots of IFN-γ staining in gated CD8 T cells. Percent positive indicated. *Bottom*, Total numbers of CD8^+^IFN-γ^+^ cells per spleen. Results are mean number ± SEM (*n* = 4 mice/group) from one experiment. *p<0.05 (WT mice vs knockout) as determined by Student’s *t* test. Similar results were obtained in three separate experiments.

To assess the impact of HVEM deficiency on the generation of CD8 T cell memory, VACV-reactive CD8 T cell populations were tracked through the production of IFN-γ following stimulation with VACV peptide epitopes at 40 days post-infection. VACV-infected WT mice contained high frequencies of memory CD8 T cells specific for all epitopes examined, regardless of whether infection was with high or low inoculum of VACV-WR (**Figure 4A**). In contrast to the primary response, irrespective of epitope specificity or viral inoculum, the accumulation of VACV-specific memory CD8 cells in HVEM−/− mice was reduced by an average of 60 to 80% (**Figure 4A**). Detailed kinetics experiments indicated that HVEM is primarily active during the expansion (day 7) and contraction (day 7–14) phases of the response with little or no effect seen during the early maintenance phase (days 14–40) of memory cells (**Figure 4B**). Significantly, 210 days post infection, both the percentage and frequency of persisting tetramer (**Figure 4C**) and IFN-γ positive (**Figure 4D**) memory VACV-reactive CD8 T cells in the spleen was 3–10 fold greater in WT mice compared with HVEM−/− mice. Together, these results show that HVEM controls the ability of VACV-specific CD8 cells to accumulate in order to form a large cytokine-competent memory pool.

### T cell Intrinsic Expression of HVEM Contributes to their Accumulation in Response to VACV Infection

**Figure 5:**
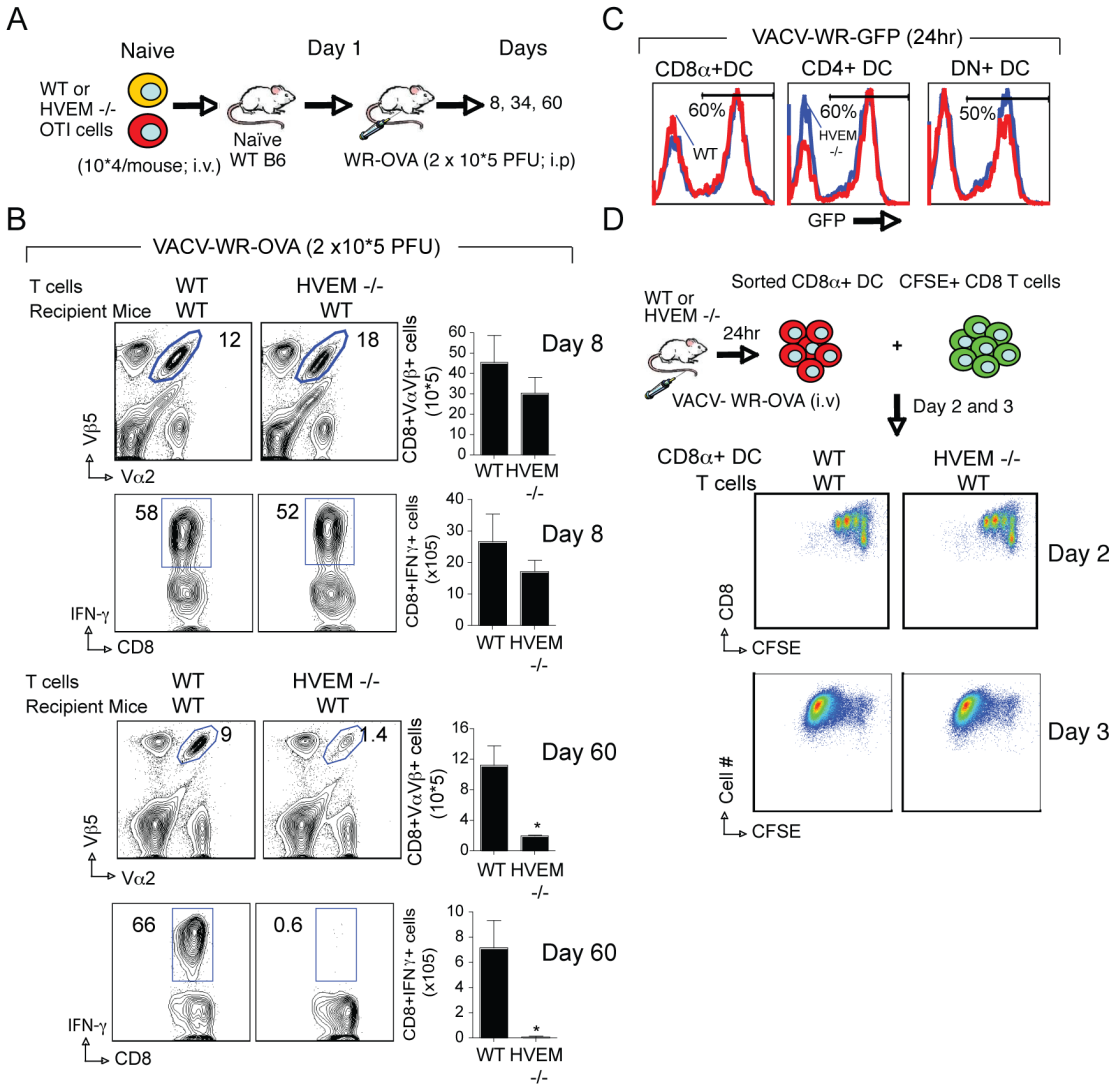
T cell–expressed HVEM is required for accumulation of large populations of memory CD8 T cells. (A) Experimental scheme. Naïve OVA-specific CD8 T cells were purified from WT or HVEM^−/−^ OT-I mice. CD8 T cells (>99% CD8^+^Vα2^+^Vβ5^+^; 1 x10^4^/mouse) were injected i.v. into naive WT mice. One day later, recipient mice were infected i.p. with recombinant VACV-WR expressing full-length OVA (VACV-WR-OVA; 2×10^5^ PFU/mouse). (B) On the indicated days, CD8 T cell expansion (Day 8; *Top panels*) and late (Day 60; *Bottom panels*) memory formation were analyzed by tracking the transgenic TCR. Contour plots, representative co-staining for IFN-γ after gating on Vα2+Vβ5+ CD8 cells. Percent positive indicated. *Right*, Total numbers of CD8^+^Vα2^+^Vβ5^+^ and CD8^+^Vα2^+^Vβ5^+^IFN-γ^+^ cells per spleen after stimulation with OVA SINFEKEL peptide. Results are mean number ± SEM (*n* = 4 mice/group) from one experiment. *p<0.05 (WT mice vs knockout) as determined by Student’s *t* test. Similar results were obtained in two additional experiments. (C) Purified splenic dendritic cell (DC) populations from WT or HVEM^−/−^ mice were cultured with recombinant VACV-WR expressing GFP in vitro. Twenty-four hours later, GFP expression in CD8α^+^, CD4^+^, and CD8α/CD4-double negative (DN) DC populations was examined. Blue line, HVEM−/− DCs; red line, WT DCs. Percentages of GFP-positive cells are indicated. (D) WT or HVEM^−/−^ mice were infected i.v. with VACV-WR-OVA. Twenty-four hours later, splenocytes were harvested and sorted for CD11c^+^CD8^+^ DC and then cultured with naive CFSE-labeled WT OT-I cells. On day 2 and 3, division of OT-I cells was examined. *Top*, Experimental scheme; *Middle*, representative dot plots of CFSE dilution on day 2 after gating on CD8^+^Vα2^+^Vβ5^+^ cells; *Bottom*, representative dot plots of CFSE dilution on day 3 after gating on CD8^+^Vα2^+^Vβ5^+^ cells. Results are the mean number ± SEM (*n* = 4 mice/group) from one experiment. Similar results were obtained in three separate experiments.

As HVEM is expressed on multiple cell types, we sought to show that HVEM was required directly by CD8 T cells responding to VACV infection. OVA-specific, HVEM-deficient CD8 T cells from OT-I TCR transgenic mice were purified and transferred into naïve WT recipients and then infected with rVACV-WR-OVA (**Figure 5A**). HVEM−/− OT-I cells expanded vigorously, and accumulated similar to WT cells by day eight (**Figure 5B**). This result closely mimicked the primary expansion of CD8 T cells from HVEM−/− mice infected with high dose (2×10^5^ PFU) of VACV-WR. However, when the ‘early’ memory populations remaining at day 34 were examined, the numbers of Ag-specific memory CD8 T cells were reduced by 65 to 70% in the absence of HVEM (not shown). Likewise, on day 60 post infection, there were few memory cells detectable in WT mice receiving HVEM-deficient OT-I cells (**Figure 5B**). This result suggests that HVEM specifically regulates survival of effector CD8 T cells after encounter with virus and consequently increases the number of cells that transition into the memory pool.

To test whether the impaired priming of CD8 T cells might also be related to defective antigen-presenting cell function during the initial stages of infection, a recombinant VACV-WR expressing GFP (rVACV-WR-GFP) was used to infect different DC populations in vitro. We focused on DC as they have previously been shown to be responsible for priming naive CD8 T cells to VACV [40], [41]. Twenty-four hours after *in vitro* infection, there was no difference observed in the capacity of any subset of HVEM−/− DC to be infected by rVACV-WR-GFP (**Figure 5C**). Next, the antigen presenting capacity of HVEM−/− CD8α^+^ DCs was examined following intravenous infection with rVACV-WR-OVA. Accordingly, one day after infection, CD8α^+^ DCs were purified from the spleen of WT and HVEM−/− mice and examined for their ability to induce proliferation of naive CFSE-labeled WT OT-I cells directly ex vivo (**Figure 5D**). WT OT-I CD8 T cells co-cultured with HVEM−/− DC proliferated to OVA at a rate comparable to those cultured with WT DC *in vitro* (**Figure 5D**). These data show that the absence of HVEM in DCs does not limit the expansion of naïve CD8 T cells driven by VACV infection and that any impaired activity was not due to defective antigen-presentation. Thus, HVEM expressed on a CD8 T cell is required for survival of effector T cells and formation of a large population of cytokine-competent memory cells during infection with VACV.

### Reduced Accumulation of VACV-specific Memory CD8 T Cells in BTLA^−/−^ Mice

**Figure 6:**
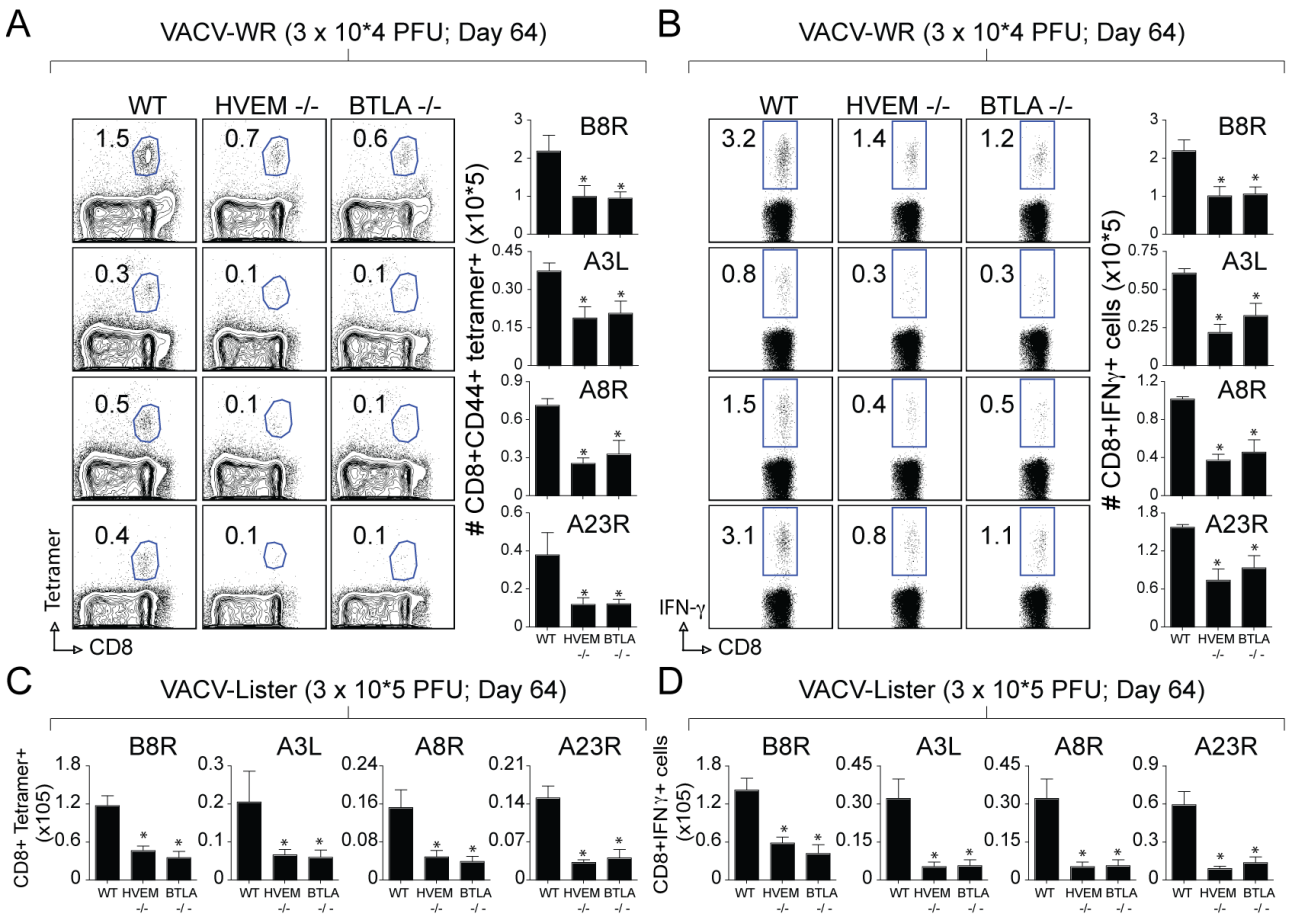
Impaired generation of CD8 memory cells to both dominant and subdominant VACV epitopes in BTLA-deficient mice. Groups of C57BL/6 WT, BTLA-deficient (BTLA^−/−^), or HVEM −/− mice were infected i.p. with the indicated inoculums of VACV-WR (A and B) and VACV-Lister (C and D). At day 64, splenocytes were harvested and stimulated with the indicated VACV peptides and CD8 T cell numbers and functionality was assessed by tetramer and intracellular IFN-γ staining. (A) Representative plots of B8R, A3L, A8R, and A23R tetramer staining, gating on CD8 cells, are shown. Percentages of activated tetramer^+^ CD8 T cells (CD8^+^CD44^+^B8R^+^) are indicated. (A) *Right*, Total numbers of CD8^+^CD44^+^tetramer^+^ cells per spleen (B) Representative plots of IFN-γ staining in gated CD8^+^CD62L^Low^ T cells. Percent positive indicated. *Right*, Total numbers of CD8^+^IFN-γ^+^ cells per spleen. Results are mean number ± SEM (*n* = 4 mice/group) from one experiment. *p<0.05 (WT mice vs gene-knockout) as determined by Student’s *t* test. Similar results were obtained in two separate experiments.

Recent studies have indicated that BTLA may act as an activating ligand that delivers a positive co-signal through HVEM to enhance T cell survival [42], [43]. To test whether BTLA was involved in the development of VACV-specific memory CD8 T cells, we infected WT and BTLA−/− mice with VACV-WR. VACV-reactive memory CD8 T cell responses were significantly reduced in BTLA−/− mice compared with WT mice, based on analyses of percentages and absolute numbers of tetramer positive CD8 T cells (**Figure 6A**), as well as the percentages and absolute numbers of IFN-γ producing cells CD8 T cells (**Figure** **6B**). Differences in the numbers of WT versus BTLA−/− CD8 T cells in the lungs paralleled those in the spleen (not shown), indicating that differences in the infected organs were not a result of differential trafficking and localization. Analysis of infectious virus produced in the ovaries revealed significant differences in the time course of primary clearance in WT versus HVEM- and BTLA-deficient mice after low dose infection correlating with the absolute numbers of virus specific CD8 T cells (**Figure S3**).

Recently we showed that the strong T cell memory elicited to the virulent virus strain VACV-WR was dependent on two TNFR family interactions, OX40 and CD27, whereas the weaker memory to the highly attenuated strain VACV-Lister was largely independent of these two molecules. To determine if this differential use of a stimulatory receptor applied to other similar molecules, HVEM was examined. In striking contrast to OX40 and CD27, at day 64, HVEM still contributed considerably to the weaker memory CD8 populations generated to VACV-Lister (**Figure** **6C and 6D**). Significantly, BTLA−/− mice showed a similar defect in generating virus-specific memory CD8 T cells. These data further show the importance of HVEM and BTLA to drive enhanced CD8 T cell memory especially in situations where the extent of exposure to viral antigen might be limited.

### Intrinsic Expression of BTLA in T cells is Dispensable for Generation of Virus-specific Effector and Memory CD8 T cells

**Figure 7:**
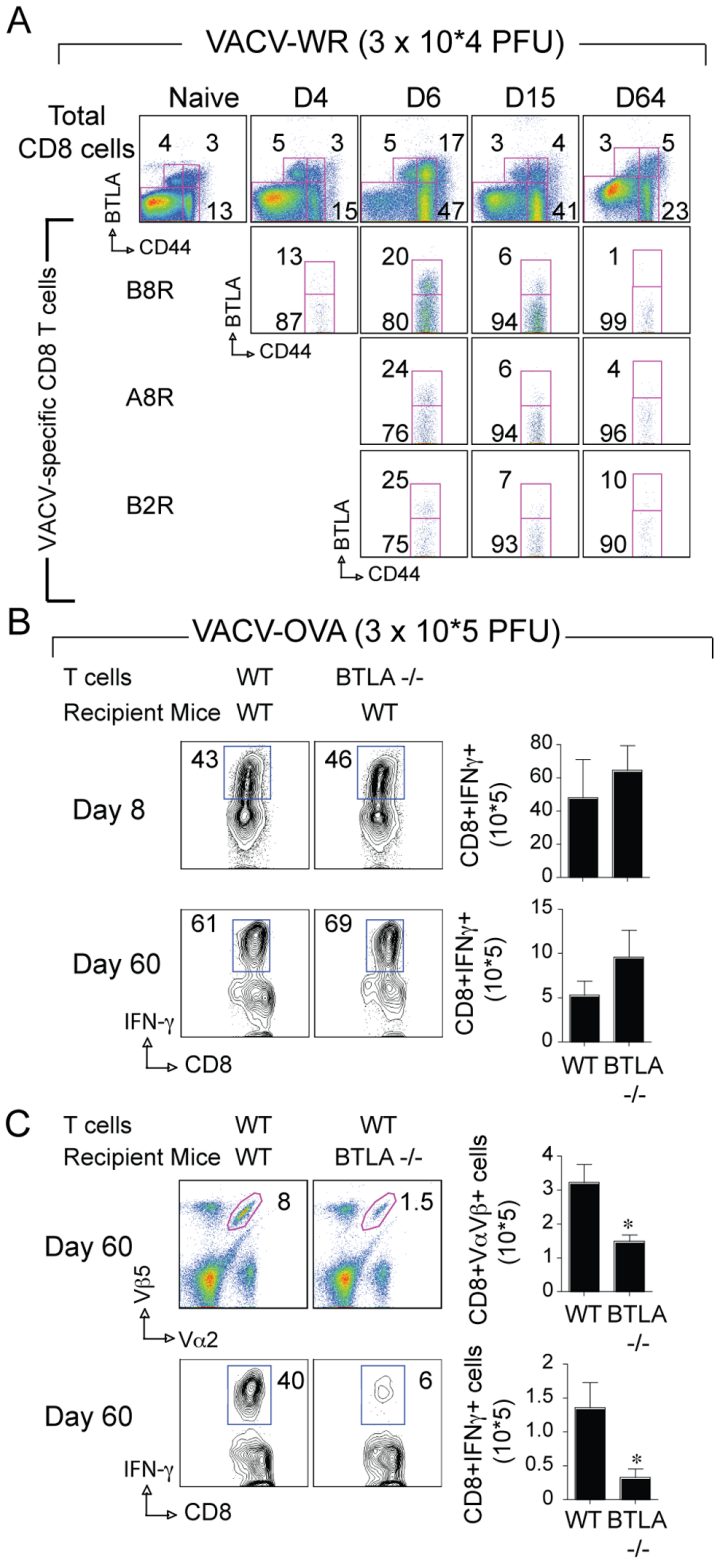
BTLA expressed on a non-T cell is required for accumulation of large populations of virus-specific CD8 T cells. (A) Groups of C57BL/6 wild-type (WT) mice were infected i.p. with VACV-WR (3×10^4^ PFU/mouse). Uninfected (naïve) mice were used as controls. On days 4, 6, 15, and 64 post-infection splenocytes were harvested and stained for CD8, CD44, VACV-specific tetramers (B8R, A8R, or B2R), and BTLA. Representative plots of BTLA staining on total CD8 T cells and tetramer (B8R, A8R, and B2R) positive cells after gating on CD8 cells. The numbers in each plot indicate the percentage of total CD8 T cells (CD44_low_ and CD44_high_) or tetramer-positive cells that stained for BTLA. (B and C) Naïve OVA-specific CD8 T cells were purified from WT or BTLA^−/−^ OT-I mice. CD8 T cells (>99% CD8^+^Vα2^+^Vβ5^+^; 1 x10^4^/mouse) were injected i.v. into naive WT (B) or BTLA−/− (C) mice. One day later, recipient mice were infected i.p. with recombinant VACV-WR expressing full-length OVA (VACV-WR-OVA; 2×10^5^ PFU/mouse). (B) On the indicated days, CD8 T cell expansion (*Top panel*), and memory formation (*Bottom panel*) were analyzed by tracking the transgenic TCR. Contour plots, representative costaining for IFN-γ after gating on Vα2+Vβ5+ CD8 cells. Percent positive indicated. *Right*, Total numbers of CD8^+^Vα2^+^Vβ5^+^IFN-γ^+^ cells per spleen after stimulation with SINFEKEL peptide. Results are mean number ± SEM (*n* = 4 mice/group) from one experiment. *p<0.05 (WT mice vs knockout) as determined by Student’s *t* test. Similar results were obtained in two additional experiments.

BTLA can be expressed on activated T cells, as well as B cells and dendritic cells [15]. VACV-specific CD8 T cells expressed BTLA as detected using the pan-specific antibody 6F7 [25], [35]. A small percentage (3–5%) of CD44^intermediate^ and CD44^high^ CD8 T cells in the spleen expressed BTLA. In contrast, CD44^low^ naive CD8 T cells did not express BTLA without antigen exposure (**Figure 7A**). Between day 4 and day 6 post-infection, BTLA surface expression was induced on 20–25% of B8R-, A8R-, and B2R-tetramer positive cells, decreased by day 15, and was nearly undetectable by day 64 after infection (**Figure 7A**). These results suggest that BTLA expression on CD8 T cells is controlled primarily during the initial phase of T cell activation. Moreover, the rapid modulation of BTLA expression in virus-specific CD8 T cells, which peaked on day 6 and down-regulated by day 15, suggests that BTLA can be readily available to some antigen-specific CD8 T cells during acute phase of VACV infection.

The impaired CD8 T cell response in BTLA−/− mice may reflect an intrinsic role for BTLA in T cells. To examine whether BTLA was required in T cells we transferred WT or BTLA−/− OT-I cells into WT mice and infected the recipient mice with rVACV-WR-OVA (**Figure 7B**). CD8 T cells from BTLA−/− OT-I mice mounted a normal effector (Day 8) and memory (Day 60) response compared with wt cells (**Figure 7B**). These T cells expanded normally upon viral infection and produced comparable levels of virus-specific effector cytokines (**Figure 7B**). This result demonstrates that BTLA expression is not required in T cells for the generation of VACV-specific effector and memory CD8 T cells, and implicates BTLA expression in non-T cells is required for memory generation.

### BTLA Expression in the Host is Required for Development of Memory in Response to VACV Infection

To determine whether BTLA expressed in a non-T cell population contributed to the defect observed in BTLA−/− mice, we transferred WT OT-I CD8 T cells into BTLA−/− hosts, and one day later infected the mice with rVACV-WR-OVA (**Figure 7C**). Strong expansion of OVA-specific wt OT-I CD8 T cells with IFN-γ-producing memory CD8 T cells. In striking contrast, very few IFN-γ-producing memory CD8 T cells were generated in BTLA −/− hosts. These data show that the absence of BTLA in the host environment limits the formation of a large population of memory cells in response to VACV infection. This phenotype closely mimicked the results of HVEM−/− OT-I cells transferred into WT hosts suggesting that BTLA expressed in the host, likely CD8α^+^ DC was required for the generation of CD8 T cell memory.

**Figure 8:**
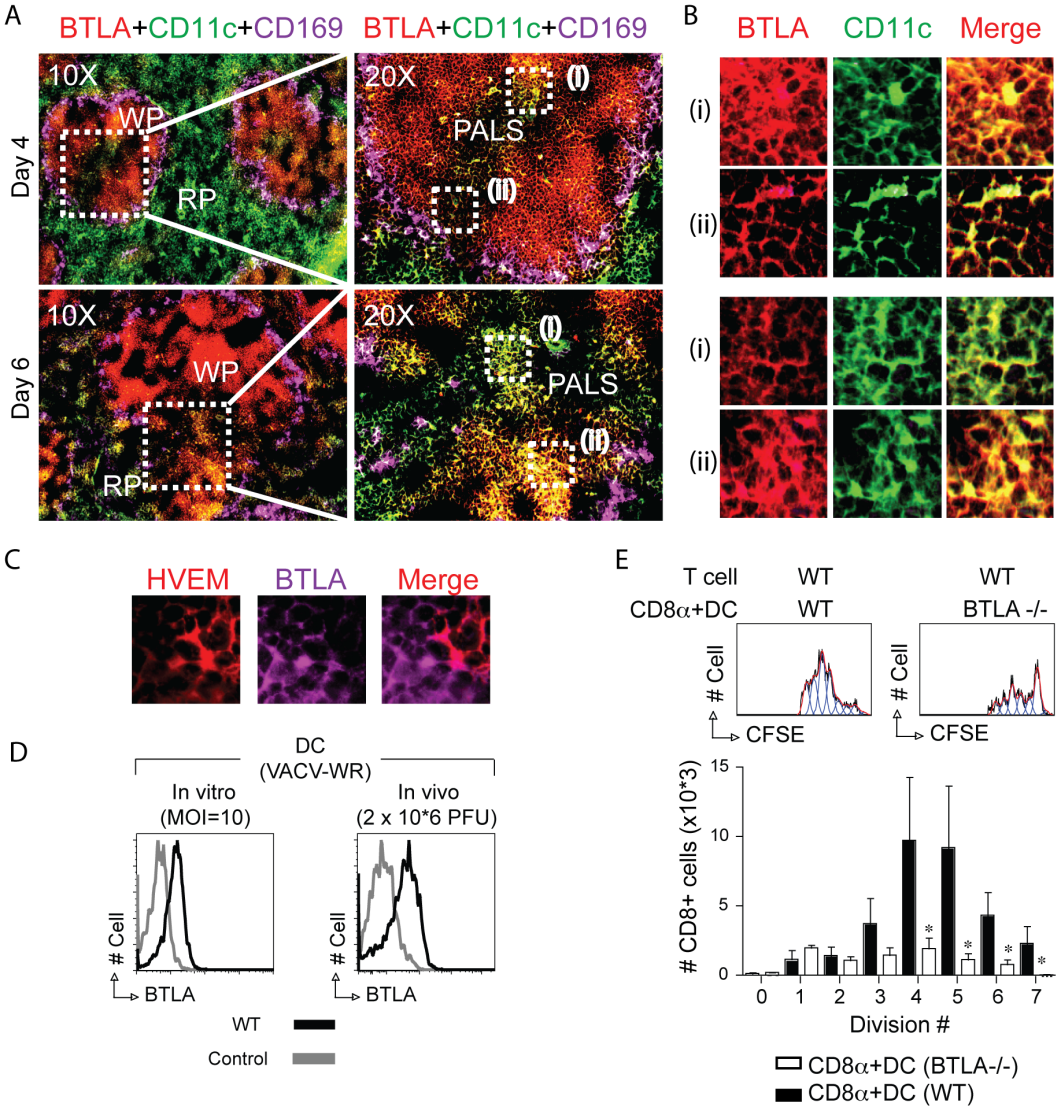
BTLA expressed on CD8α+ DCs contributes to accumulation of large populations of virus-specific CD8 T cells. (A–C) Groups of C57BL/6 wild-type (WT) mice were infected i.p. with VACV-WR (3×10^4^ PFU/mouse). Spleen was harvested on the indicated days after VACV-WR infection. Frozen sections were stained with rat anti-mouse BTLA-PE, CD169-APC for marginal zone macrophages, and CD11c-FITC for dendritic cells. The images were captured by 10×/20×/40× objectives using EVOS *fl* inverted microscope. WP, white pulp; PALS, perilymphatic sheath; RP, red pulp. Note small clusters of BTLA+ dendritic cells in the PALS. (B) CD11C and BTLA staining from the indicated areas in A (*right panels (i) and (ii)*). Colocalization signal is indicated as yellow/orange. (C) Splenic tissue 4 days post infection. Magnified view of HVEM+ cells in contact with BTLA+ cells in the PALS region of splenic white pulp. (D) BTLA expression on DCs 24 hr after in vitro (*left panel*) or in vivo (*right panel*) infection with VACV-WR. (E) WT or BTLA^−/−^ mice were infected i.v. with VACV-WR-OVA. Twenty-four hours later, splenocytes were harvested and sorted for CD11c^+^CD8α^+^ DC and then cultured with naive CFSE-labeled WT OT-I cells. Three days later, division of OT-I cells was examined. Representative histograms of CFSE dilution after gating on CD8^+^Vα2^+^Vβ5^+^ cells. (F) Absolute numbers of CD8^+^Vα2^+^Vβ5^+^ cells that accumulated after the indicated cell division. Results are the mean number ± SEM (*n* = 4 mice/group) from one experiment. Similar results were obtained in two separate experiments.

To extend these observations, we monitored the localization of BTLA+ DC in the spleen by microscopic inspection of cryosections stained with CD11c, CD169, and BTLA, (Fig. 1, A and B, and data not shown). Splenic DC are located in the T cell zone, the red pulp, the bridging channels, and in the MZ surrounding the follicle. CD8α^+^ DCs exclusively reside in the T cell zone. By day 4 and 6 PI, large numbers of CD11C+BTLA+ cells could be detected as discrete clusters in the T cell zones (PALS) of the splenic WP (**Figure 8A and 8B**). These CD11C+BTLA+ cells were often found in close contact with HVEM+ cells (**Figure 8C**). Upregulation of BTLA on CD8α^+^ DC was further confirmed by flow cytometry analysis of cells after vitro and in vivo infection with VACV-WR (**Figure 8D**).

Next we determined whether BTLA expression on CD8α^+^ DCs contributes to activation of antigen-specific CD8 T cells. By CFSE analysis, after 3 days, there was no difference in the number of cell divisions in WT OT-I CD8 T that were co-cultured with BTLA−/− CD8α^+^ DC that were purified from a VACV-WR-OVA infected host (**Figure 8E**). However, in total approximately 5–10 fold fewer T cells accumulated after each division (**Figure 8F**). Although this does not rule out the contribution of BTLA expressed on other cell types, it does suggest that the interaction of DC expressed BTLA with HVEM expressed in T cells participates in controlling the accumulation of memory T cells to viral antigen.

### HVEM Controls the Differentiation of CD8 T cells that Protect Against Lethal VACV Infection

**Figure 9:**
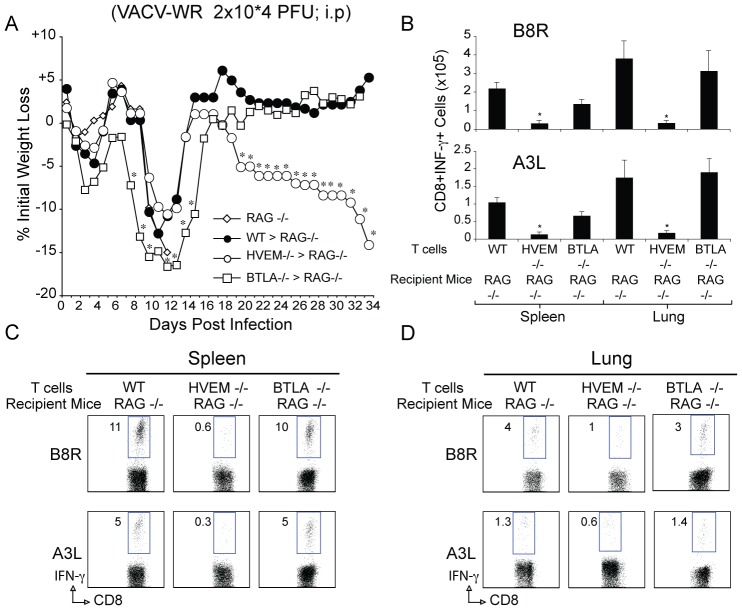
HVEM-deficient CD8 T cells fail to protect against VACV-infection. Ten million highly purified polyclonal WT, HVEM−/−, or BTLA−/− CD8 T cells were adoptively transferred into RAG^−/−^ mice. One day later, mice were infected i.p. with VACV-WR (2×10^4^ PFU/mouse). Animals were weighed daily and euthanized if weight loss was greater than 25% body weight. (A) Mean % of initial body weight from indicated numbers of mice. Thirty-four days after infection, virus-specific CD8 T cells were assessed in the spleen (B and C) and lungs (B and D) by intracellular cytokine staining after ex vivo stimulation with VACV B8R or A3L peptide. Data are representative plots of IFN-γ staining in gated CD8 T cells, with percent positive indicated, or total numbers ± SEM of CD8+IFN-γ+ T cells per tissue from four individual mice. *p<0.05 (WT vs gene-knockout treated) as determined by Student’s *t* test. Similar results were obtained in two separate experiments.

We addressed whether the role of HVEM in driving VACV-reactive CD8 T cells was important for protection from the virus. We utilized mice deficient in T and B cells (Rag −/−) to separate antiviral CD8 T cell activity from protective antibody responses. After infection with VACV-WR, Rag −/− mice exhibit weight loss and death within 12–14 days (**Figure 9A**). Adoptive transfer of naïve polyclonal CD8 T cells into Rag −/− recipients can afford protection against death in this model (**Figure 9A**). An increased number of CD8 T cells were transferred to limit homeostatic proliferation, allowing us to examine the VACV antigen-induced response in a non-transgenic system. Weight loss (15% to 20%) as a measure of disease was observed in these mice, suggesting that they were not fully immune (**Figure 9A**). Rag −/− mice receiving BTLA−/− CD8 T cells also lost weight and then fully recovered between days 14 and 18, mirroring the response of WT CD8 T cells. However, HVEM−/− CD8 T cell recipients failed to control the virus late in the response and eventually all animals succumbed to the infection. On day thirty-four, the overall number of B8R- and A3L-tetramer reactive (not shown) and IFN-γ-producing VACV-specific CD8 T cells generated from HVEM−/− donor populations was strongly reduced in the spleen and lungs (**Figure 9B–D**).

A notable difference between the WT B6 and the Rag −/− mice used here is the kinetics of CD8 T cell expansion and contraction, which is considerably delayed in the Rag transfer model. As shown in **Figure S4** low numbers of WT VACV-specific CD8 T cells were detected on day 7 post-infection. These cells then expanded by ∼10-fold between days 7 and 15 and then contracted between days 15 and 34 (compare **FigureS S4A** and **S4B**; and **Figure 9B**). This delay we think is due to the low precursor frequency of virus-specific CD8 T cells transferred into the Rag −/− mice (Ten million naïve polyclonal CD8 T cells were adoptively transferred into Rag −/− recipients). Significantly, the kinetics of CD8 T cell expansion mirrored the pattern observed with regards to the weight loss recovery and viral clearance.

Absolute numbers of HVEM−/− CD8 T cells were strongly reduced at day 7 postinfection and this translated into fewer cells surviving to day 15 and during early memory (day 34; **Figures** **9B–D**). We observed little evidence of accelerated contraction in the HVEM −/− CD8 T cells between days 15 and 34 compared with WT T cells. WT, BTLA −/− and HVEM−/− T cells underwent a similar fold expansion between days 7 and 15 and this mirrored to recovery of weight loss **(** **Figure 9A** **)** and a 10-fold reduction in viral titers (**Figure S4; compare 4A and 4B)**. Between days 15 and 34 WT and BTLA−/− CD8 T cells were present at sufficiently high enough numbers to almost completely clear the virus from the ovaries. HVEM−/− CD8 T cells also contributed to viral clearance during this period but significant levels (∼100-fold greater than WT recipients) of virus remained in the ovaries at day 34. Therefore, after the contraction phase of the response, HVEM-deficient CD8 T cells fail to accumulate in high enough numbers to sustain the response against vaccinia virus and this failure likely contributes to the weight loss observed between days 15 and 34. These results are similar to the response of HVEM−/− OT-I cells and implicating HVEM regulates survival of effectors and formation of memory T cells. Thus, HVEM signaling in VACV-specific CD8 T cells dictates the frequency of protective VACV-specific memory CD8 T cells elicited during viral infection.

## Discussion

HVEM serves as a molecular switch activating both stimulatory and inhibitory pathways crucial for T cell homeostasis. HVEM activates NF-kB after binding the canonical TNF-related molecule LIGHT, serving as a costimulatory receptor during activation of T cells [23], [24]. HVEM also functions as a ligand for the Ig superfamily member BTLA, which serves as an inhibitory signaling receptor limiting T cell function [14], [15], [19]. With VACV, our results highlight a previously unappreciated role for the HVEM-BTLA co-signaling system in anti-viral CD8 T cell immunity. We show that BTLA functions as an unconventional trans-activating ligand for HVEM that strongly promotes anti-viral effector T cell survival and memory formation. These findings illustrate that the HVEM-BTLA trans-complex forms a bidirectional signaling system that may serve as either an inhibitory and pro-survival system for CD8 T cells.

The present study revealed that BTLA functions not only as an inhibitory receptor, BTLA could also, in the context of a live virus infection, serve as a trans-activating ligand for HVEM. This investigation provides a physiological context to recent biophysical and cellular models for HVEM-BTLA signaling pathways [42], [43]. HVEM-BTLA trans-cosignaling system strongly influenced the survival of VACV-specific CD8 T cells and was necessary for the generation of large populations of memory cells. Based on published studies in autoimmune disease models we anticipated that VACV-specific BTLA−/− CD8 T cells would have augmented responses. Unexpectedly however, BTLA−/− CD8 T cells did not show any signs of hyper responsiveness to dominant and subdominant VACV peptide epitopes at both the effector or memory phases of the response. Instead through a series of experiments, we found that T cell-autonomous expression of HVEM is necessary for continued accumulation of virus-specific effector CD8 T cells and optimal generation of memory. Similar to CD8 T cells lacking HVEM, WT CD8 T cells failed to accumulate effectively in a BTLA-dificient environment upon VACV challenge. Moreover, WT CD8 T cells co-cultured with VACV-infected BTLA−/− CD8α^+^ DCs failed to survive and accumulate over time. These results clearly illustrate that BTLA acts as a trans-activating ligand that delivers pro-survival signals through HVEM expressed on CD8 T cells. Interestingly, this exact function was also proposed for HVEM during infection with the intracellular bacteria *Listeria monocytogenes* (see companion paper by Steinberg *et al*.), substantiating the idea that HVEM-BTLA co-signaling system could mediate similar functional outcomes in CD8 T cells responding to different pathogen derived antigens.

Recent biophysical and cellular studies demonstrated that BTLA, CD160 and HSV gD, like LIGHT, activate HVEM to initiate a TRAF2-dependent serine kinase cascade that specifically promotes RelA activation and cell survival [42], [44]. BTLA−/− CD4 and CD8 T cells survived poorly following anti-CD3 activation in vitro, however, substitution with a soluble surrogate of BTLA, BTLA-Fc, activated NF-kB and rescued BTLA−/− T cells from cell death [43], [44]. Notably, this function of BTLA occurred in LIGHT- and CD160-sufficient T cells, suggesting HVEM-BTLA trans-activating pathway is independent of these other HVEM ligands [44]. Therefore, the simplest model, which is supported by our results, indicates that HVEM-BTLA derived co-signals are required for survival of CD8 T cells late during their differentiation at the time when VACV antigen is encountered. Without these HVEM dependent signals many of the responding T cells will die, rather than expand and survive to form the high frequency pools of effector and memory cells required for effective host defense.

An important observation is that the HVEM-BTLA *trans*-cosignaling system is strongly active in the development of CD8 T cells that protect against lethal VACV infection. In this regard, HVEM behaves similar, but independently, to TNFR family members, CD27 and OX40 [39], [45], which further provide key pro-survival signals during T cell activation via TRAF, NF-kB, and AKT dependent pathways [7], [10]. Our results highlighted here reveal an important aspect of TNFR/TNF superfamily usage during anti-viral immunity. OX40 and CD27 drive the generation of a high frequency of protective memory CD8 T cells only when VACV replication occurred over extended periods, or when the inoculum of virus was increased significantly, also translating to a greater viral load over time [45]. By contrast, HVEM-BTLA interactions were predominantly active during low-dose infections or when viral antigens were limiting due to attenuation of the virus as in the case with VACV-Lister. This result was further illustrated by the fact that memory CD8 T cells responding to several subdominant VACV peptide epitopes (A3L, A8R, A23R, and B2R) increasingly relied on HVEM-BTLA derived pro-survival signals as compared with populations responding to the immunodominant epitope B8R. A prerequisite for efficient control of viral variants is that populations of effector and memory cells are generated that exhibit diversity in the range of viral antigens they recognize. In this regard, we recently demonstrated that all of the subdominant epitopes examined here are as effective as B8R in conferring protection against a highly lethal VACV challenge [46]. Thus, an intriguing interpretation of our results is that HVEM-BTLA derived signals serve to increase the diversity of the CD8 T cells responding to VACV, particularly in situations where OX40 and CD27 may not be optimally active.

At present we do not know why anti-viral CD8 T cell responses in conditions of high virulent/doses of virus might be dependent on OX40 and CD27 while low virulent/doses might be HVEM dependent but we are actively pursuing these lines of investigations. The simplest explanation may be that the inflammatory environment created by viruses that show little or no replication (or during low dose infections) does not provide necessary co-signals that are needed to allow upregulation of CD27 ligand (CD70) or OX40 ligand (OX40L) on antigen presenting cells. Another possibility is that under low dose infection CD70 and OX40L are induced but expression is not high enough or sustained long enough for them to impact on CD8 T cells. Perhaps a certain length of time of OX40L and CD70 mediated signaling is required to allow optimal survival of effector CD8 T cells. In support of some of these ideas we have preliminarily data showing that infection with the highly virulent vaccinia virus WR strain results in rapid and sustained up-regulation of OX40L and CD70 on CD8α+ DCs whereas infection with the attenuated VACV-Lister does not (Salek-Ardakani unpublished data). In striking contrast, BTLA is readily induced on multiple DC subsets after infection with VACV-WR and VACV-Lister. Therefore, we think that virulence factors and inflammatory environment may critically dictate the expression of molecules like OX40L, CD70, and BTLA on APCs and hence their activity during vaccinia virus infection.

BTLA and HVEM can also form *cis*-complex [24], [43], [47], with BTLA directly blocking trans-activation of HVEM by all its Ig-related ligands [43]. Naïve T cells coexpress HVEM and BTLA on the cell surface at approximately 1∶1 ratio, with only the ecto domain of BTLA required for interference with ligand binding. This finding was proposed as a potential mechanism responsible for maintaining naïve T cells in a resting state [43] by limiting the influence of its ligands expressed in the surrounding microenvironment. Paradoxically, more recent evidence indicated BTLA could also, under certain conditions, function as a costimulatory receptor by initiating survival signals for effector T cells. Accordingly, BTLA−/− T cells reactive to alloantigens in a graft vs host disease (GVHD) setting expanded initially but failed to survive over time [34], [36]. Similarly in a CD4 T cell transfer model of colitis, BTLA−/− T cells transferred into Rag−/− hosts failed to accumulate, with the reduced number of effector T cells in the recipients ultimately affecting disease progression [29]. In the colitis model, effector CD4 T cell survival required the presence of HVEM-BTLA cis-complex [43]. HVEM does not activate NF-kB dependent signals in the cis-complex, suggesting BTLA may provide the intrinsic survival mechanism in the cis-complex [43]. Taken together, these studies illustrate that the role of HVEM as an inhibitory or stimulatory co-signaling ligand in T cells is critically affected by the physiological context of receptor ligand expression and possibly by the nature of immunizing antigen. Thus, HVEM predominantly serves as a ligand to deliver inhibitory BTLA cosignals in autoimmune and inflammatory disease models that involve priming with non-replicating antigen in an artificial inflammatory environment. By contrast, in GVHD and certain colitis models HVEM serves as an activating ligand to deliver pro-survival BTLA co-signals.

In summary, our studies together with others demonstrate that the physiologic context (non-replicating antigen vs live infection) of HVEM-BTLA *trans*-complex critically determines signaling outcome: signaling in trans can provide inhibitory or pro-survival co-signalings for activated T cells, and cis-interactions in T cells limits responsiveness to signals from adjacent cells. This demonstrates the plasticity of this intriguing co-signaling system in utilizing similar mechanisms to achieve different outcomes.

## Materials and Methods

### Ethics Statement

This study was carried out in strict accordance with the recommendations in the Guide for the Care and Use of Laboratory Animals of the animal.

Welfare Act and the National Institutes of Health. All animal protocols were approved by the Institutional Animal Care and Use Committee (IACUC) of the La Jolla Institute for Allergy and Immunology, San Diego (OLAW Assurance # A3779-01).

### Mice

8–12 wk-old female C57BL/6 mice were purchased from the Jackson Laboratory (Bar Harbor, ME). HVEM−/− and BTLA−/− on the BL/6 background were bred in house. OVA-specific HVEM- and BTLA-deficient CD8 T cells were produced by crossing HVEM- and BTLA-knockout mice to OT-I TCR transgenic mice.

### Peptides and Tetramers

Peptides used were: B8R (20–27; TSYKFESV), A3L (270–227; KSYNYMLL), A8R (189–196; ITYRFYLI), B2R (54–62; YSQVNKRYI), A23R (297–305; IGMFNLTFI) [38], [39]. MHC/peptide tetramers for the epitope B8R (20–27; TSYKFESV)/H-2K^b^, conjugated to allophycocyanin, were obtained from the National Institutes of Health Tetramer Core facility (Emory University, Atlanta, GA).

### Viruses

VACV Western Reserve (VACV_WR_) strain was purchased from ATCC (Manassas, VA), grown in HeLa cells, and titered on VeroE6 cells [48].

### Virus Infections

Mice were infected intraperitonealy (i.p.) with different inoculums of VACV as before [39], [48]. Effector responses were analyzed between days 4 and 15 post-infection, while memory responses were analyzed 40 or more days after infection, after restimulation in vitro with VACV peptides.

### VACV-titer Assay

Tissues from individual mice were homogenized, and sonicated for 0.5 min with a pause every 10 seconds using an ultrasonic cleaner 1210 Branson (Danbury, CT). Serial dilutions were made and the virus titers were then determined by plaque assay on confluent VeroE6 cells.

### CD8 T cell Isolation from Spleen

For adoptive transfer experiments, 1×10^4^ naive WT OT-I CD8 T cells were purified from spleens of indicated naive donor mice with MACS technology (Miltenyi Biotec) and transferred into WT non-transgenic B6 WT, HVEM −/−, or BTLA −/− mice. One day later, mice were infected i.p. with recombinant VACV-WR expressing full-length OVA protein (rVACV-OVA, 2×10^5^ PFU/mouse) or PBS as indicated [40], [41]. OT-I expansion and effector formation were detected by FACS staining of transgenic TCR α- and β-chains after gating on CD8 T cells and in some cases after restimulating in vitro with OVA (SINFEKL) peptide.

### CFSE Labeling of Transgenic CD8 T cells

Splenocytes were obtained from CD8^+^ OT-I TCR-transgenic mice and purified using positive selection with MACS beads (Miltenyi Biotec). Enriched cells contained 99% specific CD8^+^ TCR-transgenic T cells. These were labeled with CFSE (Molecular Probes) by incubating 10^7^ purified cells per ml with 5 µM CFSE for 10 min at 37°C. Cells were then washed three times in HBSS containing 2.5% FCS.

### Analysis of in vitro Activation of Naive T cells by DC

A total of 5×10^4^ CD8^+^-purified CFSE-labeled TCR-transgenic cells was added to 1.25×10^4^ fluorescence- activated cell-sorted DC in 200 µl of RPMI 1640 containing 10% FCS, 50 µM 2-ME, 2 mM **l**-glutamine, 100 U/ml penicillin, and 100 µg/ml streptomycin (complete medium) in 96-well V-bottom plates (Costar; Corning). Each culture was performed in duplicate. Cultures were analyzed for proliferation after 48 or 72 h as indicated. Cells were stained with anti-CD8-PerCP (53–6.7; BD Pharmingen), anti-Vα2-PE (B20.1; BD Pharmingen), and anti-Vβ5-allophycocyanin cells from the entire well were analyzed for proliferation by flow cytometry.

### Flow Cytometry

Cytokine production in T cells was performed as previously described [41], with some modifications. Briefly, after lysing RBC, splenocytes from infected mice were resuspended in RPMI 1640 medium (Life Technologies) supplemented with 10% FCS (Omega Scientific), 1% l-glutamine (Invitrogen), 100 mg/ml streptomycin, 100 U/ml penicillin, and 50 mM 2-ME (Sigma-Aldrich). One to 2×10^6^ cells were plated in round-bottom 96-well microtiter plates in 200 µl with medium or the indicated VACV peptides at 1 µg/ml for 1 h at 37°C. GolgiPlug (BD Biosciences) was then added to the cultures according to the manufacturer’s instructions and the incubation was continued for 7 h. Cells were stained with anti-CD8 (PerCP; 53–6.7) and CD62L (PE; MEL-14), followed by fixation with Cytofix/Cytoperm (BD Biosciences) for 20 min at 4°C. Fixed cells were subjected to intracellular cytokine staining in BD Perm/Wash buffer for 30 min at 4°C. Anti-IFN-γ (allophycocyanin; XMG1.2) was obtained from eBiosience and used at a 1/100 dilution. Samples were analyzed for their proportion of cytoplasmic cytokines after gating on CD8^+^CD62L^low^ T cells by a FACSCalibur flow cytometer using CellQuest (BD Biosciences) and FlowJo software (Tree Star).

### Immunofluorescence Studies

At various times post infection with VACV spleen was harvested and immediately snap frozen in OCT on dry ice. The OCT embedded 5 µm thick cryo-sections of spleen were cut by Microm HM 505E cryostat and prepared on super frost glass slides for immunofluorescence microscopy. Each slide of cryo-sections was washed with 1 ml phosphate buffered saline (PBS) and non specific sites were blocked by incubating sections with 2% horse serum for 30 min at 4°C. After blocking, the sections were washed with cold PBS and incubated overnight in the dark at 4°C with rat anti-mouse HVEM PE or APC conjugated (dilution 1∶100), rat anti-mouse BTLA PE or APC conjugated (dilution 1∶100), rat anti-mouse CD3 PE conjugated (dilution 1∶100), rat anti-mouse B220 PE or FITC conjugated (dilution 1∶100 or 1∶50 respectively), rat anti-mouse CD11c FITC conjugated (dilution 1∶50), rat anti-mouse CD169 APC or PE conjugated (dilution 1∶100) as indicated in the figure legends. Sections were washed three times with PBS, mounted with cytoseal and covered with glass coverslip. The stained sections were observed and analyzed at wavelength 488 nm for FITC (green), 543 nm for PE (red) and 647 for APC (purple) labeling, using a EVOS *fl*, (Advanced Microscopy Group inverted immunofluorescence microscope) and images were captured by 4×, 10×, 20× and 40× objectives, keeping all the conditions of microscope and settings of software identical for all treatments and controls.

### Statistics

Statistical significance was analyzed by Student’s *t* test. Unless otherwise indicated, data represent the mean ± SEM, with *p*<0.05 considered statistically significant.

## Supporting Information

Figure S1 **Localization T and B cells in the spleen of naive mice.** Spleen was harvested from naïve mice. Frozen sections were stained with with rat anti-mouse antibodies against CD3 (PE) for T cells, CD169 (FITC) for marginal zone (MZ) metallophilic macrophages and B220 (PE) for B cells. The images were captured by 4× and 10× objective using EVOS fl inverted microscope. Arrows indicate splenic marginal zones (MZ; Top right panel; green) and MZ bridging channels (MZBC: bottom right panel). B cell follicles (F) were identified by B220 (red channel). Perilymphatic sheath (PALS) were identified by CD3 (red channel). RP, Red pulp; WT, white pulp.
(TIF)

Figure S2 **HVEM expression on OVA-specific CD8 T cells (OT-1) responding to VACV-WR-OVA infection.** One 10^5^ naïve congenically marked (CD45.2) WT OT-1 cells were adoptively transferred into naïve WT (CD45.1) mice. One day later, mice were infected i.p. with VACV-WR-OVA. On the indicated days, OT-I CD8 T cells were analyzed for HVEM expression. Left column; Representative plots of costaining for CD45.2 and CD8; *Middle column*, plots of HVEM staining, gating on CD45.2 negative (endogenous) CD8 T cells; *Right column*, plots of HVEM staining, gating on CD45.2 positive (adoptively transfered) CD8 T cells. Numbers indicate the percentage of CD8+HVEM+/− T cells. Similar results were obtained in three separate experiments.
(TIF)

Figure S3 **HVEM- and BTLA-deficient mice fail to clear virus after low dose infection with VACV-WR.** WT, HVEM- and BTLA-deficient mice were infected i.p. with VACV-WR (3 x 10^3^ PFU/mouse). At day 14 postinfection, ovaries were removed and VACV-titers were determined as described in Materials and Methods.
(TIF)

Figure S4 **HVEM-deficient CD8 T cells fail to protect against VACV-infection.** Ten million highly purified polyclonal WT, HVEM−/−, or BTLA−/− CD8 T cells were adoptively transferred into RAG−/− mice. One day later, mice were infected i.p. with VACV-WR (2 x 10^4^ PFU/mouse). On day 7 (A) and day 15 (B) virus-specific CD8 T cells were assessed in the spleen by intracellular cytokine staining after ex vivo stimulation with VACV B8R, A3L, A8R, and A23R peptides. Total numbers ± SEM of CD8+IFN-γ+ T cells per spleen from four individual mice. *p<0.05 (WT vs gene-knockout treated) as determined by Student’s t test. Similar results were obtained in two separate experiments. (C) On the indicated days post infection, ovaries were removed and VACV-titers were determined as described in Materials and Methods.
(TIF)
