# Supplementary figures and images for: CD8 T Cell Memory to a Viral Pathogen Requires Trans Cosignaling between HVEM and BTLA
Source: PLoS One. 2013 Oct 29;8(10):e77991. doi: 10.1371/journal.pone.0077991 (PMC3812147; doi:10.1371/journal.pone.0077991)

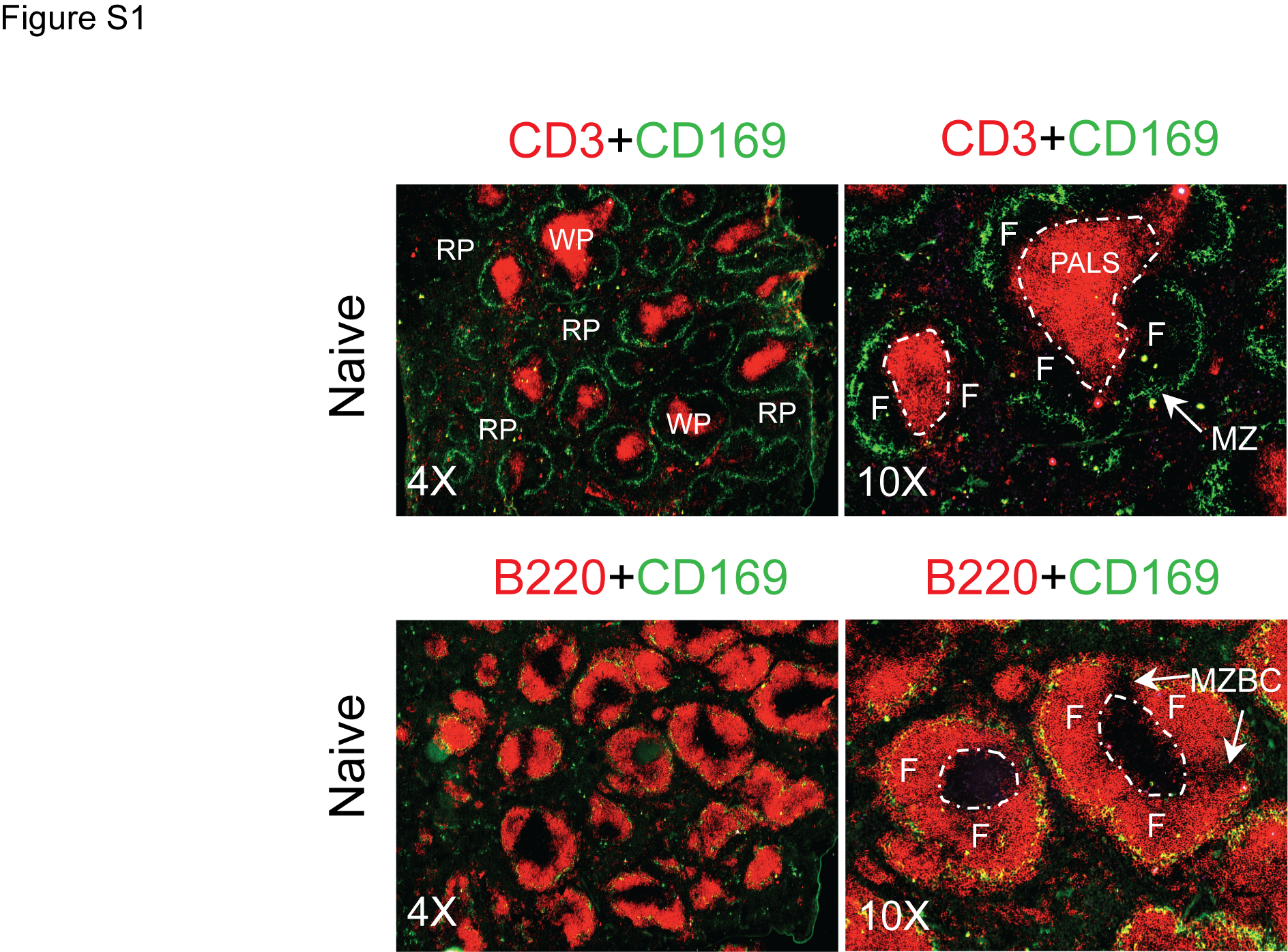

Supplement: Figure S1 — Localization T and B cells in the spleen of naive mice. Spleen was harvested from naïve mice. Frozen sections were stained with with rat anti-mouse antibodies against CD3 (PE) for T cells, CD169 (FITC) for marginal zone (MZ) metallophilic macrophages and B220 (PE) for B cells. The images were captured by 4× and 10× objective using EVOS fl inverted microscope. Arrows indicate splenic marginal zones (MZ; Top right panel; green) and MZ bridging channels (MZBC: bottom right panel). B cell follicles (F) were identified by B220 (red channel). Perilymphatic sheath (PALS) were identified by CD3 (red channel). RP, Red pulp; WT, white pulp. (TIF) [file pone.0077991.s001.tif]

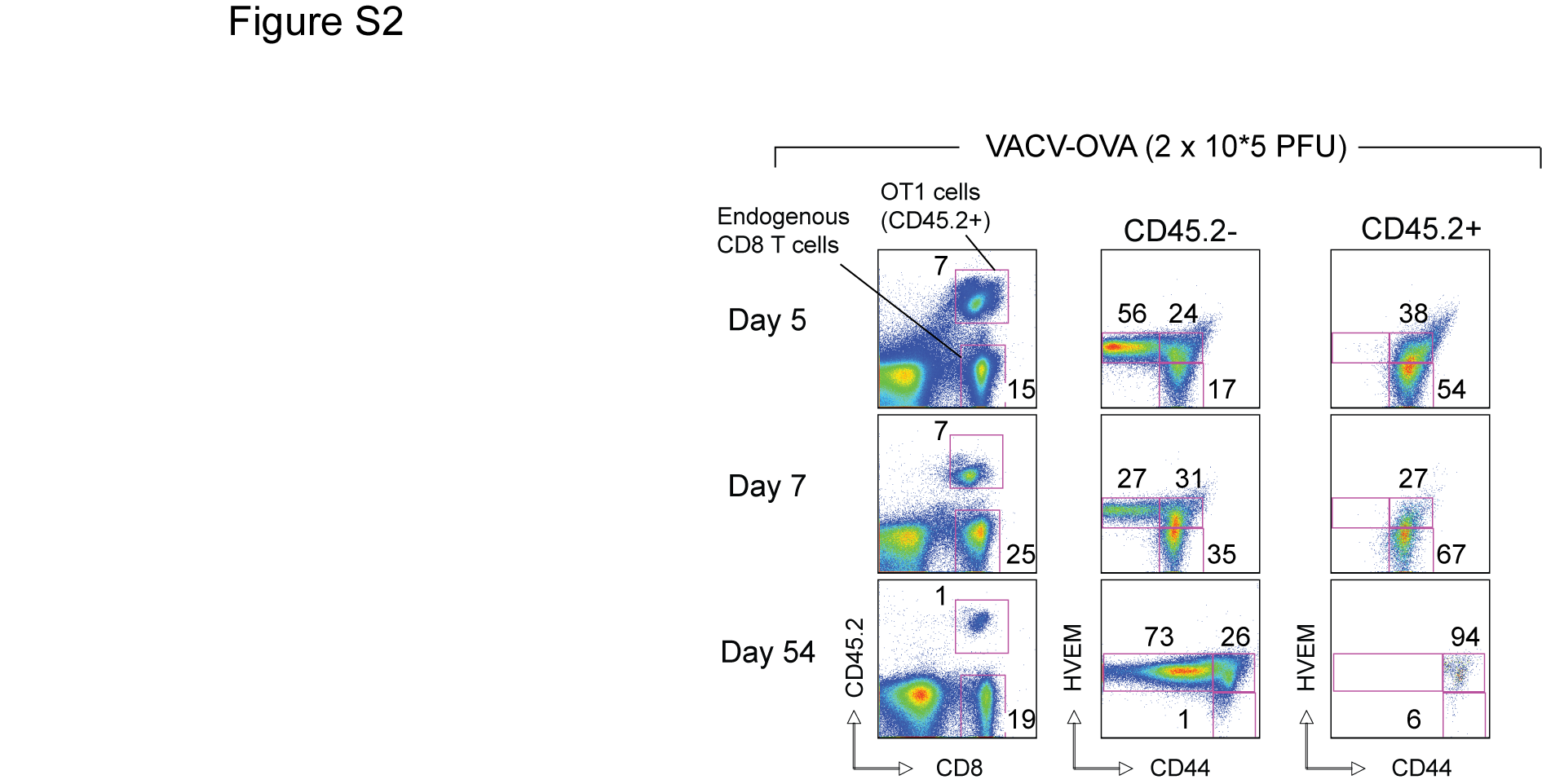

Supplement: Figure S2 — HVEM expression on OVA-specific CD8 T cells (OT-1) responding to VACV-WR-OVA infection. One 105 naïve congenically marked (CD45.2) WT OT-1 cells were adoptively transferred into naïve WT (CD45.1) mice. One day later, mice were infected i.p. with VACV-WR-OVA. On the indicated days, OT-I CD8 T cells were analyzed for HVEM expression. Left column; Representative plots of costaining for CD45.2 and CD8; Middle column, plots of HVEM staining, gating on CD45.2 negative (endogenous) CD8 T cells; Right column, plots of HVEM staining, gating on CD45.2 positive (adoptively transfered) CD8 T cells. Numbers indicate the percentage of CD8+HVEM+/− T cells. Similar results were obtained in three separate experiments. (TIF) [file pone.0077991.s002.tif]

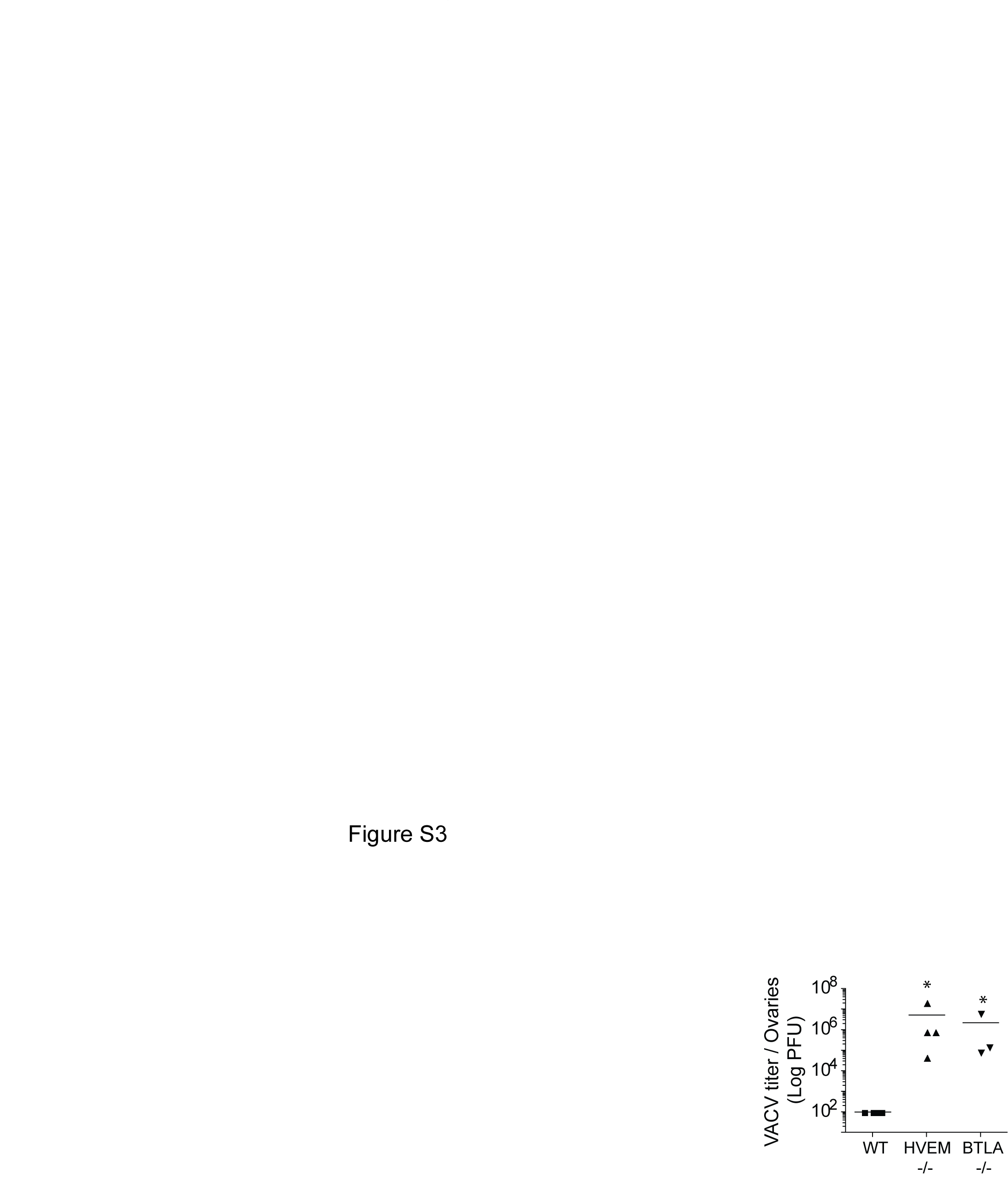

Supplement: Figure S3 — HVEM- and BTLA-deficient mice fail to clear virus after low dose infection with VACV-WR. WT, HVEM- and BTLA-deficient mice were infected i.p. with VACV-WR (3 x 10^3^ PFU/mouse). At day 14 postinfection, ovaries were removed and VACV-titers were determined as described in Materials and Methods. (TIF) [file pone.0077991.s003.tif]

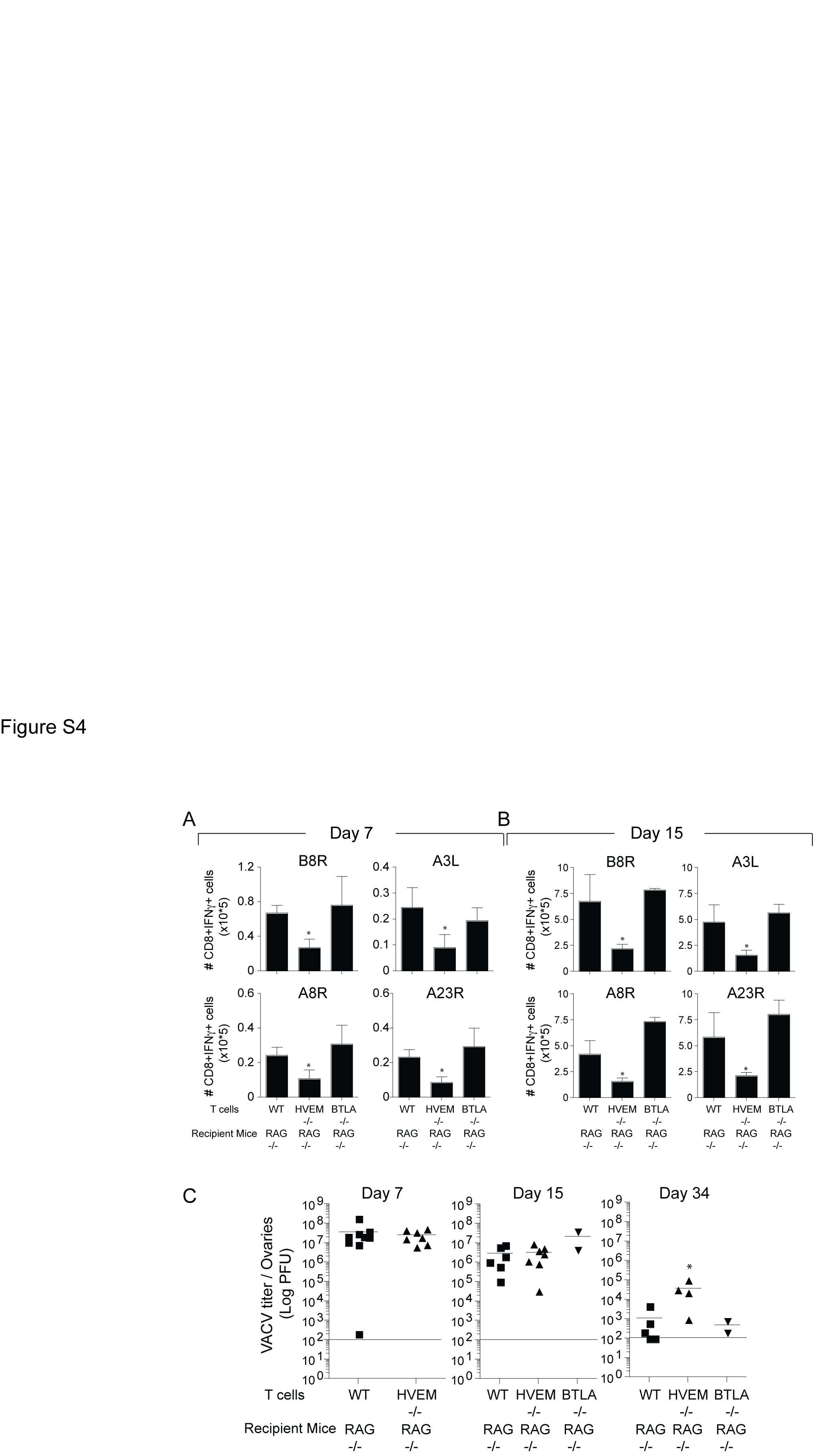

Supplement: Figure S4 — HVEM-deficient CD8 T cells fail to protect against VACV-infection. Ten million highly purified polyclonal WT, HVEM−/−, or BTLA−/− CD8 T cells were adoptively transferred into RAG−/− mice. One day later, mice were infected i.p. with VACV-WR (2 x 10^4^ PFU/mouse). On day 7 (A) and day 15 (B) virus-specific CD8 T cells were assessed in the spleen by intracellular cytokine staining after ex vivo stimulation with VACV B8R, A3L, A8R, and A23R peptides. Total numbers ± SEM of CD8+IFN-γ+ T cells per spleen from four individual mice. *p<0.05 (WT vs gene-knockout treated) as determined by Student’s t test. Similar results were obtained in two separate experiments. (C) On the indicated days post infection, ovaries were removed and VACV-titers were determined as described in Materials and Methods. (TIF) [file pone.0077991.s004.tif]
